# Allelic Variation of Cytochrome P450s Drives Resistance to Bednet Insecticides in a Major Malaria Vector

**DOI:** 10.1371/journal.pgen.1005618

**Published:** 2015-10-30

**Authors:** Sulaiman S. Ibrahim, Jacob M. Riveron, Jaclyn Bibby, Helen Irving, Cristina Yunta, Mark J. I. Paine, Charles S. Wondji

**Affiliations:** 1 Vector Biology Department, Liverpool School of Tropical Medicine, Liverpool, United Kingdom; 2 Department of Chemistry, University of Liverpool, Liverpool, United Kingdom; The University of North Carolina at Chapel Hill, UNITED STATES

## Abstract

Scale up of Long Lasting Insecticide Nets (LLINs) has massively contributed to reduce malaria mortality across Africa. However, resistance to pyrethroid insecticides in malaria vectors threatens its continued effectiveness. Deciphering the detailed molecular basis of such resistance and designing diagnostic tools is critical to implement suitable resistance management strategies. Here, we demonstrated that allelic variation in two cytochrome P450 genes is the most important driver of pyrethroid resistance in the major African malaria vector *Anopheles funestus* and detected key mutations controlling this resistance. An Africa-wide polymorphism analysis of the duplicated genes *CYP6P9a* and *CYP6P9b* revealed that both genes are directionally selected with alleles segregating according to resistance phenotypes. Modelling and docking simulations predicted that resistant alleles were better metabolizers of pyrethroids than susceptible alleles. Metabolism assays performed with recombinant enzymes of various alleles confirmed that alleles from resistant mosquitoes had significantly higher activities toward pyrethroids. Additionally, transgenic expression in *Drosophila* showed that flies expressing resistant alleles of both genes were significantly more resistant to pyrethroids compared with those expressing the susceptible alleles, indicating that allelic variation is the key resistance mechanism. Furthermore, site-directed mutagenesis and functional analyses demonstrated that three amino acid changes (Val^109^Ile, Asp^335^Glu and Asn^384^Ser) from the resistant allele of *CYP6P9b* were key pyrethroid resistance mutations inducing high metabolic efficiency. The detection of these first DNA markers of metabolic resistance to pyrethroids allows the design of DNA-based diagnostic tools to detect and track resistance associated with bednets scale up, which will improve the design of evidence-based resistance management strategies.

## Author Summary

Scale up of Long Lasting Insecticide Nets has massively reduced malaria mortality across Africa. However, resistance to pyrethroid insecticides in malaria vectors threatens its continued effectiveness. Here, we established that allelic variation in two CYP450s is the most important driver of pyrethroid resistance in the major African vector *Anopheles funestus* and detected key mutations controlling this resistance. The duplicated P450s *CYP6P9a* and *CYP6P9b* are directionally selected across Africa with alleles segregating according to resistance phenotypes. Alleles from resistant mosquitoes present significantly higher metabolic activities toward pyrethroids compared with alleles from susceptible mosquitoes. Furthermore, transgenic flies over-expressing resistant alleles of both genes were significantly more resistant to pyrethroids. Three amino acid changes from the resistant *CYP6P9b* allele are the key pyrethroid resistance mutations which induce high metabolic efficiency. The detection of these first DNA markers of metabolic resistance to pyrethroids allows the design of diagnostic tools to detect and track resistance.

## Introduction

Despite the recent decrease in malaria mortality (47%) [1], the disease remains a serious public health burden in the tropical world, with 584,000 deaths globally in 2013, of which 90% occurred in WHO African region, and mostly in children under the age of 5. Malaria control relies heavily on the use of insecticide-impregnated LLINs and indoor residual spraying (IRS) [2]. Unfortunately, resistance to insecticides, especially pyrethroids (the only class approved by WHO for LLINs [3]), in major malaria vectors such as and *An*. *funestus* [4–6] and *An*. *gambiae* [7, 8] is threatening to derail these intervention tools [9]. *An*. *funestus* is widely, geographically distributed across Sub-Saharan Africa [10], and it has high vectorial capacity in some places surpassing even that of *An*. *gambiae* [11]. It reaches maximal abundance in the dry season when the density of *An*. *gambiae* and *An*. *arabiensis* have declined, thereby extending the period of malaria transmission [12]. Cases of resistance to pyrethroid, carbamate and organochlorine insecticides are increasingly reported in *An*. *funestus* populations across Africa [4, 5, 13–15]. It is imperative to design and implement suitable resistance management strategies to limit the impact of such resistance (WHO, 2012). One prerequisite is the development of appropriate diagnostic tools to facilitate the monitoring of insecticide resistance at an early stage, in order to inform control programs of the best course of action to take. However, the design of DNA-based diagnostic tools requires a thorough understanding of the molecular basis of the resistance.

To date, efforts to characterise mechanisms of resistance in malaria vectors have implicated knockdown resistance (*kdr*) and metabolic resistance through elevated expression of resistance genes, especially cytochrome P450s [6, 8] as the two major mechanisms conferring resistance to pyrethroids. No *kdr* mutation has been reported in the voltage-gated sodium channel of *An*. *funestus* [2]; pyrethroid resistance is therefore mainly metabolic. However, despite the numerous reports of implications of over-expressed P450s in pyrethroid resistance, the detailed molecular mechanisms through which they confer pyrethroid resistance in mosquitoes remain largely uncharacterised. It also remains unclear whether mechanisms other than P450 over-expression are also involved in resistance, for example, allelic variation with changes in the coding sequences through mutations of key amino acid residues or *cis* and/or *trans* mutations which could impact gene regulation [16]. Recent observation of polymorphism variations for the two most important pyrethroid resistance genes, *CYP6P9*a and *CYP6P9b*, in the malaria vector *An*. *funestus* [17, 18] suggests that this mosquito species is an excellent candidate to assess the impact of allelic variations of resistance genes on pyrethroid resistance. Recently, for the glutathione S-transferase gene, *GSTe2*, in *An*. *funestus*, a single point mutation (Leu^119^Phe) was detected and established to confer DDT resistance [15]. If such causative mutation(s) could be identified for pyrethroid resistance mediated by P450 genes, it will facilitate the design of DNA-based diagnostic tools to easily detect and track such resistance in field populations.

Knowledge of insect P450s’ preferential sites of metabolism of insecticides can facilitate the design of highly specific synergist inhibitors against the detoxification genes. For example, in insects and other organisms the 4´ spot of the phenoxybenzyl ring have been shown to be the preferential sites of hydroxylation [19, 20], which is followed by hydrolysis to generate intermediates including alcohols and acids which could easily be conjugated. On the other hand fluorogenic probes are increasingly used to identify inhibitors of P450s, and as diagnostic compounds to establish the degree of binding of insecticide substrates to P450s [21, 22].

In this study, sequence characterisation of *CYP6P9a* and *CYP6P9b* across Africa detected important polymorphism variations. *In silico* predictions, *in vitro* and *in vivo* functional characterisation tools were then applied to demonstrate that allelic variation in *CYP6P9a* and *CYP6P9b* is the key molecular change through which *An*. *funestus* mosquitoes acquire high resistance to pyrethroid insecticides. Furthermore, site-directed mutagenesis coupled with *in vitro* functional characterisation of the mutant recombinant proteins detected three major amino acid changes responsible for high pyrethroid metabolising efficiency of *CYP6P9b* from resistant populations of *An*. *funestus s*.*s*., compared to susceptible allele. This will allow the design of DNA-based diagnostic assay to detect and track this resistance in field populations in Africa.

## Results

### Africa-wide patterns of genetic variability of *CYP6P9a* and *CYP6P9b*

#### Analysis of cDNA polymorphism

**Fig 1:**
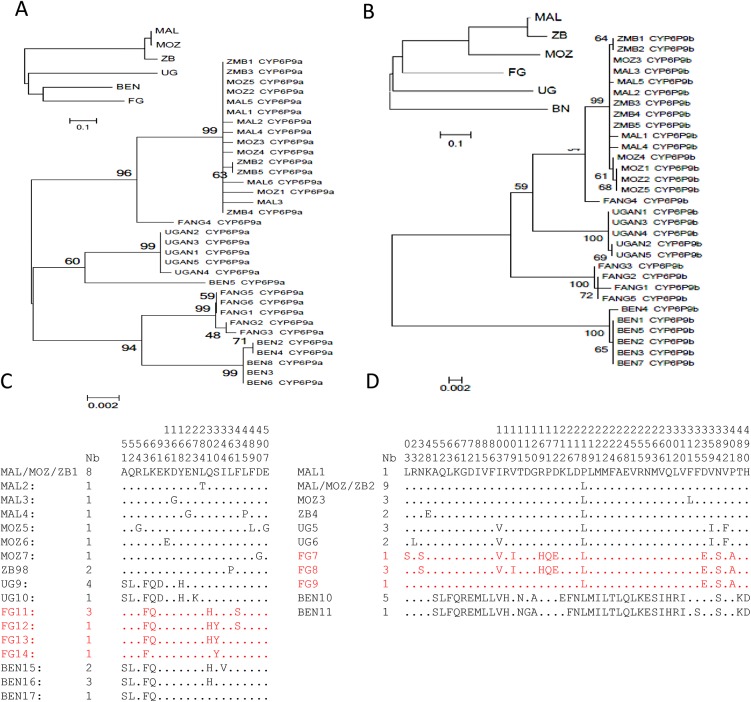
Schematic representation of haplotypes of *An*. *funestus CYP6P9a* and *CYP6P9b* from resistant populations and the susceptible strain FANG, across Africa. (A) and (B) shows maximum likelihood tree of *CYP6P9a* and *CYP6P9b* cDNA sequences which forms clades specific to each phenotype. The small trees on the upper left of the maximum likelihood trees indicate the *Nst* genetic distances; (C) and (D) shows the polymorphic positions for both *CYP6P9a* and *CYP6P9b* amino acid sequences, respectively. A number is given to each haplotype preceded by country of origin (BEN, UGAN, MAL, MOZ, ZMB or FANG, for Benin, Uganda, Malawi, Mozambique, Zambia and FANG strains, respectively). The column Nb stands for the number of individual mosquitoes sharing a haplotype.

Analysis of the polymorphisms patterns of full-length cDNA sequences of *CYP6P9a* and *CYP6P9b* (1527bp for both) from different regions of Africa revealed a relative homogeneity within each geographical region but significant variations between haplotypes from different regions of Africa, and also the laboratory susceptible population (FANG). *CYP6P9a* has 17 haplotypes and 74 polymorphic sites of which 22 were non-synonymous, with the highest polymorphism observed in Benin and FANG (S1 Table). *CYP6P9b* has 11 haplotypes across Africa and 138 polymorphic sites the bulk of which were contributed from larger variations in the Benin and FANG alleles compared with Uganda and southern African alleles (Malawi, Mozambique and Zambia). Overall, the relative genetic homogeneity in each region is shown by (i) the fact that haplotypes from each country and region cluster together on the maximum likelihood phylogenetic tree (Fig 1A and 1B), notably those from the three southern African countries; (ii) the fact that only few amino acid changes (5 to 7) are observed within regions in contrast to the higher number of replacements between them (22 to 51) (S1 Table). A predominant haplotype is also observed for each gene in each region, notably across southern Africa (Fig 1C and 1D), where the predominant allele for each gene is the one previously associated with pyrethroid resistance when comparing alive (resistant) and dead (susceptible) mosquitoes after permethrin exposure in [4, 17]. Interestingly, the predominant haplotype in FANG was found exclusively in permethrin susceptible mosquitoes in southern Africa, suggesting that this allele is associated with susceptibility to pyrethroids [17]. The majority of the amino acid replacements are observed between the resistant haplotypes from southern Africa and those from FANG (Fig 1C and 1D) with the exception of *CYP6P9b* haplotype from Benin. These significant allelic variations are further supported by the neighbour-joining tree based on the *Nst* genetic distances showing high genetic distances between samples apart from those of southern Africa which are very close genetically (Fig 1A and 1B).

To determine if the allelic variations between the haplotypes could significantly impact the ability of these two genes in conferring pyrethroid resistance, a comparative analysis of the metabolic activity of all these alleles was carried out.

### Allelic variations and *in silico* analysis of *CYP6P9a* and *CYP6P9b* alleles

#### Functional mapping of key amino acid changes

In comparison to FANG, for both *CYP6P9a* and *CYP6P9b* sequences, several amino acid changes observed in field resistant alleles were mapped to important domains of these P450s (S1 and S2 Figs).

Based on the haplotype differences, a single predominant allele in each region of Africa or in the FANG was chosen for further characterisation. For *CYP6P9a*, the selected alleles were: MAL/MOZ/ZB1 (Fig 1C) for southern Africa (hereafter *MALCYP6P9a*), UG9 for East Africa (*UGANCYP6P9a*), BEN16 for West Africa (*BENCYP6P9a*), and FG11 for FANG (*FANGCYP6P9a*). For *CYP6P9b*, the selected alleles were: MAL/MOZ/ZB2 (Fig 1D) for southern Africa (hereafter *MALCYP6P9b*), UG5 for East Africa (*UGANCYP6P9b*), BEN10 for West Africa (*BENCYP6P9b*) and FG8 for the FANG (*FANGCYP6P9b*). Both *FANGCYP6P9a* and *FANGCYP6P9b* were previously shown to be present only in the pyrethroid susceptible mosquitoes in southern Africa [17] and thus, are representative of the field pyrethroid susceptible mosquitoes.

**Fig 2:**
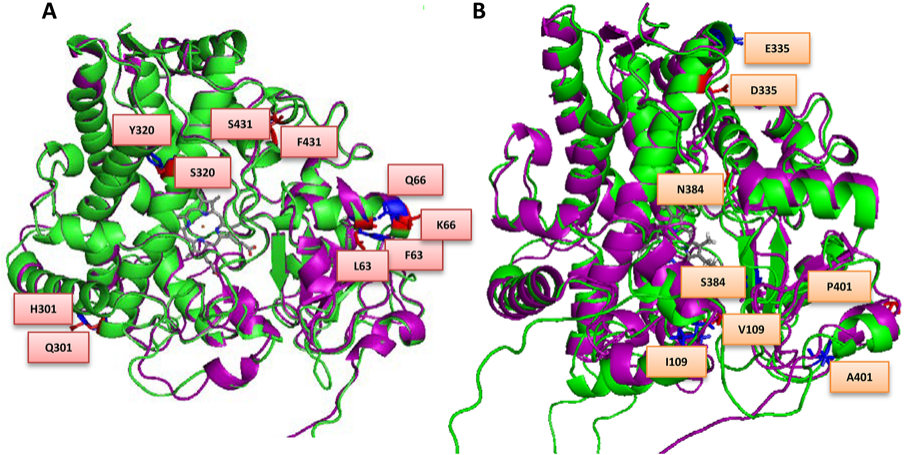
Overlay of (A) MALCYP6P9a (green helices) and FANGCYP6P9a (purple helices) and (B) MALCYP6P9b (green helices) and FANGCYP6P9b (purple helices) showing amino acid residues changes. Key residues are in stick format and those belonging to MALCYP6P9a or MALCYP6P9b are highlighted in red and annotated, while corresponding residues in FANGCYP6P9a and FANGCYP6P9b are in blue colour. Heme atoms are shown in stick format and grey.

The predicted impact of the amino acids substitutions with relevant references used for the analysis are outlined in S2 Table, and the key mutations predicted to be important for catalysis are presented in Fig 2A and 2B, for both *MALCYP6P9a* and *MALCYP6P9b* in comparison to FANG.

#### Prediction of activity of allelic variants using molecular docking

Docking simulations predicted differences in the binding conformations and affinities of pyrethroids within the active sites of the different CYP6P9a and CYP6P9b models from resistant alleles compared with models from the susceptible alleles. Productive poses were defined as docking solutions with the 4´ spot of phenoxy ring approaching the heme iron (the preferred site of hydroxylation described for insect P450s [19, 23]), or the solutions with *cis*/*trans*-methyl group approaching the heme iron (minor route of hydroxylation) if the 4´ spot is away for optimal interaction to take place. Distances of between 3-6Å were considered productive to allow for optimal van der Waals contacts with minimum overlaps [24]. Higher ChemScore values and/or lower free energy of binding predicted by the GOLD software were considered to reflect higher catalytic activity.

#### Permethrin (Type I pyrethroid) docking

For CYP6P9a, permethrin docked into the active sites of models from resistant alleles (e.g. MALCYP6P9a) productively with *trans* methyl group oriented for hydroxylation at a distance of 4.2Å from the heme iron (S3A Fig), while in FANGCYP6P9a the insecticide docked with *trans* methyl group away (6.5Å) for optimal metabolism to occur (S3B Fig). The FANGCYP6P9a produced lowest ChemScore and highest free energy of binding suggesting that the susceptible allele may possess the lowest activity toward permethrin (S3 Table).

**Fig 3:**
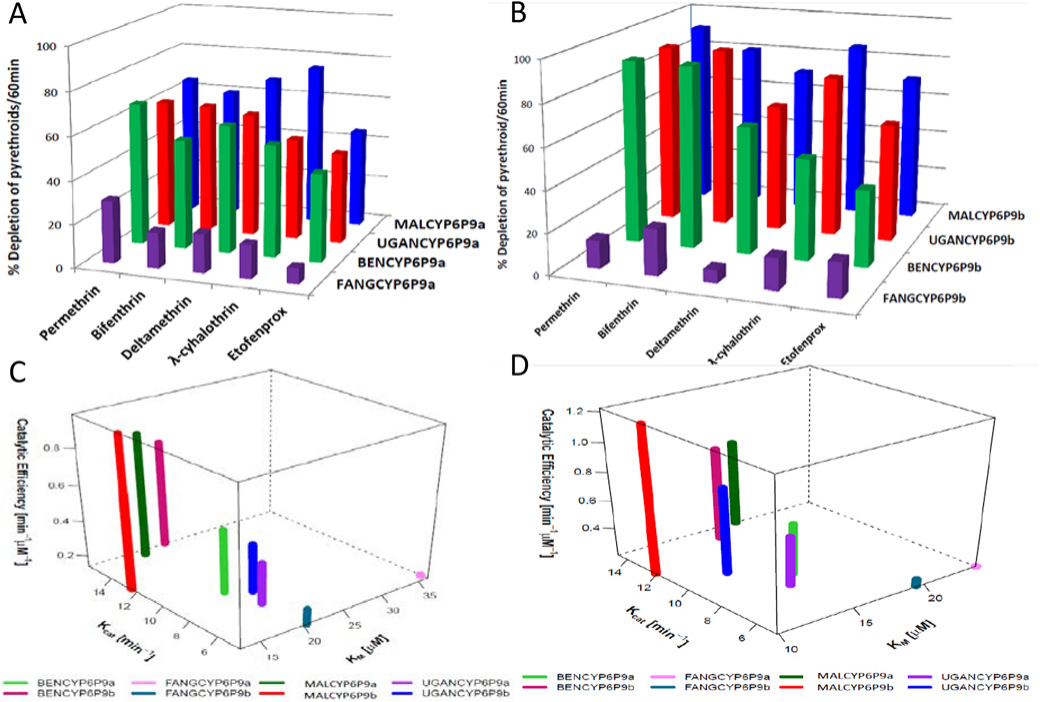
Substrates depletion assay results and kinetic profiles of recombinant CYP6P9a and CYP6P9b. Percentage depletion of 20μM pyrethroids by (A) CYP6P9a and (B) CYP6P9b proteins. Results are average of three replicates (n = 3) compared with negative control (-NADPH); (C and D) 4D plot of the kinetic constants and catalytic efficiencies of CYP6P9a and CYP6P9b metabolism of permethrin and deltamethrin, respectively.

For CYP6P9b, permethrin docked productively in the model of the resistant allele (e.g. MALCYP6P9b) with the 4ʹ spot of the phenoxy ring oriented above the heme at a distance of 3.2Å (S3C Fig). The insecticide docked unproductively in the active site of FANGCYP6P9b with dichlorovinyl groups approaching the heme, and the possible sites of attack away from the catalytic centre: *trans*-methyl group located 10.4Å from heme iron (Figs 3D and S3D). Binding parameters for permethrin in the models of the resistant alleles and FANG are provided in S3 Table.

#### Deltamethrin (Type II pyrethroid) docking

Deltamethrin docked productively in the active site of MALCYP6P9a and MALCYP6P9b with *trans* methyl group within 3.5Å distance of the heme iron in MALCYP6P9a (S4A Fig) and the 4ʹ spot of phenoxy ring exposed for hydroxylation at reasonable distance of 3.7Å from the heme iron, in MALCYP6P9b (S4C Fig). The insecticide did not bind productively in the corresponding models from FANGCYP6P9a and FANGCYP6P9b. In FANGCYP6P9a, the insecticide docked with *trans* methyl group approaching the heme (6.0Å), but the dibromovinyl group projects toward the heme (S4B Fig). In FANGCYP6P9b the 4ʹ spot of phenoxy ring is the closest site of metabolism, but is located 8.9Å from heme iron for optimal interaction (S4D Fig). Binding parameters for deltamethrin in the models of the resistant alleles and FANG are provided in S3 Table.

Moreover, *MALCYP6P9b* allele was predicted to possess highest activity towards both permethrin and deltamethrin, compared with all the alleles of *CYP6P9a* and *CYP6P9b* analysed. In addition, the docking software predicted *cis*/*trans*-methyl hydroxylation to occur as well from MALCYP6P9b-mediated metabolism of deltamethrin, leading to multiple primary metabolites.

### Comparison of substrate access channels between *CYP6P9a* and *CYP6P9b* models

#### Identification of pw2a substrates access channel

Because substrate binding and product release by P450s may impact their role in drug metabolism [25], a molecular simulation of substrate access and/or product egress routes was performed for all CYP6P9a and CYP6P9b models. The algorithm predicted that the models from the resistant and susceptible alleles of *CYP6P9a* alleles did not differ in their substrate access and/or product egress channels. In contrast, differences were observed in the channels composition and parameters between the models of resistant (S5A Fig) and susceptible (S5B Fig) alleles from *CYP6P9b*. For example, for MALCYP6P9b model, a total of 30 tunnels were predicted from the interior active site of the protein to the bulk solvent. Of these channels the first ranked with highest throughput (0.76), lowest energy cost (0.27) and which is longest (21.42Å) has its gorge lined with about 41 residues (S5A Fig). One of these residues is the Val^109^ from BʹC loop which was established to be a member of channel *pw2a* proposed to be the most common route of access and egress for most CYP450s [26, 27]. Channel *pw2a* has been established as the most energetically favourable of all the pathways observed in *CYP101* [27] and was described as a product egress route for *CYP101* and *CYP2B4*. This later property was possible because the *pw2a* is a *holey* channel (wide-open); a property made possible due to the potential motion in the FG loop and/or the BC loop. Val^109^ of SRS-1, a substitution common to all resistant alleles of CYP6P9b is located within contact distance (2.9Å) of this *pw2a* tunnel. For CYP6P9b the mutation Ile^109^Val possibly modifies the substrates accessing machinery in the resistant allele with impact on substrate recognition and/or affinity.

CAVER computed 23 tunnels for FANGCYP6P9b of which the first ranked with lower throughput (0.74), has higher cost (0.31) and is almost half short (13.91Å) (S5B Fig) compared with the first-ranked tunnel from MALCYP6P9b model. The corresponding Ile^109^ of *FANGCYP6P9b* is located 7.7Å from the tunnel and is therefore not a tunnel lining residue in all of the 23 predicted channels. This further strengthened the importance of the mutation Val^109^ in the resistant alleles of *CYP6P9b*. However, this simulation was conducted with deltamethrin only, although other substrates could access or egress differently in *CYP6P9b* or other P450s.

### Comparative assessment of metabolic activity of *CYP6P9a* and *CYP6P9b* alleles using *in vitro* functional characterisation

Having established that the allelic variations observed between *CYP6P9a* and *CYP6P9b* haplotypes induced significant differences in their 3D structures and possibly their ability to interact and metabolise pyrethroid insecticides, we next validated these observations through *in vitro* and *in vivo* characterisation experiments.

### *In vitro* expression and metabolism assays with recombinant enzymes

Recombinant proteins of the various *CYP6P9a* and *CYP6P9b* alleles were produced with optimal expression between 36–56 hours as described previously [4]. No significant differences were observed in P450 content and cytochrome P450 reductase activity between the recombinant proteins expressed from the various alleles of *CYP6P9a* and *CYP6P9b* (hereafter named resistant alleles) compared to proteins obtained from the FANG alleles (hereafter referred to as susceptible alleles) (S6A and S6B Fig). Thus, reductase content may not induce any allelic variation in metabolic activities.

#### Pyrethroid metabolism assays

Recombinant enzymes from resistant alleles of both *CYP6P9a* and *CYP6P9b* metabolize Type I (permethrin and bifenthrin) and Type II (deltamethrin and λ-cyhalothrin) pyrethroids, as well as etofenprox, with statistically significant depletion (+NADPH vs–NADPH) (S4 Table and Fig 3A and 3B). In contrast, only very low and non-significant depletions (not more than 20%) were obtained with proteins from susceptible alleles, for both genes. These results further support the observation that allelic variation in both genes is significantly impacting their ability to metabolise pyrethroid insecticides. In agreement with the docking results no major differences in pyrethroid metabolism were observed between the different field resistant alleles, although on average, proteins from the southern African alleles had higher depletion compared to corresponding proteins from other regions of Africa. For example, with *CYP6P9a* alleles, MALCYP6P9a exhibited highest activity against λ-cyhalothrin with a depletion of 75.41±1.72 significantly higher than for UGANCYP6P9a (47.74±1.5) (p<0.05). The same pattern was observed when depletion of λ-cyhalothrin and etofenprox from MALCYP6P9b was compared with values from BENCYP6P9b (p<0.05) (S4 Table). Overall, highest metabolic activities were observed from recombinant CYP6P9b proteins especially MALCYP6P9b which deplete more than 90% of permethrin and more than 80% of bifenthrin, deltamethrin and λ-cyhalothrin. *CYP6P9b* alleles consistently exhibited higher activities toward permethrin, bifenthrin and deltamethrin compared with *CYP6P9a* counterparts.

To further assess the impact of allelic variation on activity towards pyrethroids, steady-state kinetic parameters were established with permethrin and deltamethrin, for each CYP6P9a and CYP6P9b membrane. Reactions proceed via Michaelis-Menten mechanism (S7 Fig), with *K*_*m*_ values within ranges (1–50μM) previously described for binding and metabolism of pyrethroid by insect P450s [28]. For CYP6P9a with permethrin, the *K*_*cat*_ value obtained with MALCYP6P9a was the highest and 3-fold higher than the amount obtained with the FANGCYP6P9a from the susceptible allele (p<0.05) (Fig 3C and S5 Table). The *K*_*cat*_ from Benin and Uganda recombinant proteins with permethrin, were also 2-fold higher than values from FANG (BENCYP6P9a vs FANGCYP6P9a, p<0.05). In terms of affinity, the susceptible FANGCYP6P9a proteins exhibited significantly higher *K*_*m*_, two-fold the values obtained with proteins from the resistant alleles (p<0.05). These differences translated into variation in catalytic efficiencies with MALCYP6P9a exhibiting the highest efficiency, 6-fold more efficient than FANGCYP6P9a (p<0.05), while BENCYP6P9a and UGANCYP6P9a were also 3-fold higher in activity compared with FANGCYP6P9a (p<0.05).

The same pattern of allelic differences in activities was also observed with respect to *CYP6P9b* (Fig 3). BENCYP6P9b exhibited the highest activity for permethrin compared with all the other alleles, with its *K*_*cat*_ and that from MALCYP6P9b and UGANCYP6P9b 4-fold, 3-fold and 2-fold respectively higher compared to the *K*_*cat*_ obtained from FANGCYP6P9b (p<0.05) (Fig 3C and S5 Table). No major differences were observed in terms of affinity toward permethrin (*K*_*m*_) between FANGCYP6P9b and the resistant alleles from East (UGANCYP6P9b) and West Africa (BENCYP6P9b), whereas, the southern African allele (MALCYP6P9b) significantly differs by producing low, reproducible *K*_*m*_ (on average half the values obtained from FANGCYP6P9b as well as the other resistant alleles of *CYP6P9b*, p<0.05). These differences in kinetic parameters between resistant and susceptible alleles were reflected in the catalytic efficiency, with MALCYP6P9b 5-fold more efficient in permethrin metabolism than FANGCYP6P9b (p<0.05).

For deltamethrin, similar higher activities and catalytic efficiencies were obtained from the recombinant proteins expressed from the resistant alleles compared with the FANG (Fig 3D and S5 Table). Further analysis of kinetic parameters obtained with deltamethrin is discussed in more detail in *S1 Text*.

Consistent with the *in silico* predictions, the catalytic efficiencies for both pyrethroids metabolism by the alleles of *CYP6P9b* were higher than values from *CYP6P9a*. The *K*_*m*_ of the southern African allele of *CYP6P9b* was also significantly lower compared with southern African *CYP6P9a* allele (p<0.05) (S5 Table), reflecting the pre-eminence of *CYP6P9b* over *CYP6P9a* in terms of pyrethroid metabolism and conferment of resistance.

#### Fluorogenic probes assays

**Fig 4:**
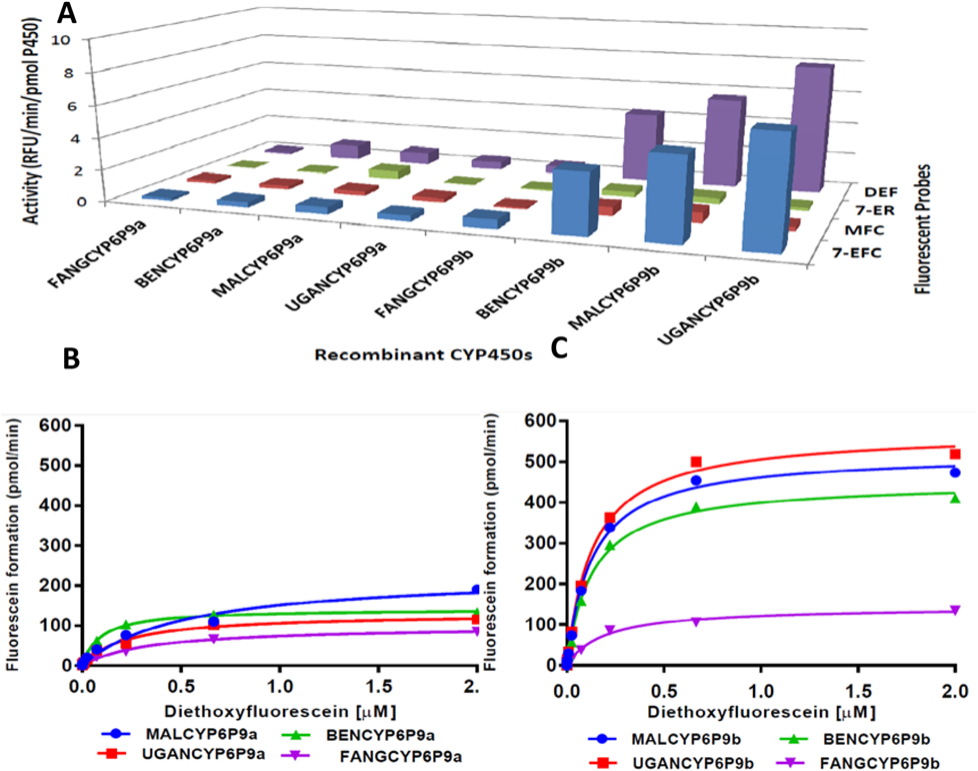
Metabolism of probe substrates by various recombinant CYP6P9a and CYP69b proteins. (A) Initial testing of O-dealkylation. The solid bars indicate average of significant turnovers of three experimental replicates compared to negative controls (-NADPH). DEF, diethoxyfluorescein; 7-ER, 7-ethoxyresorufin; MFC, 7-methoxyfluoromethylcuomarin; and 7-EFC, 7-ethoxy-4-trifluoromethylcuomarin. (B and C) Michaelis-Menten plots for recombinant (B) CYP6P9a and (C) CYP6P9b metabolism of diethoxyfluorescein.

Fluorescent probes were screened to determine whether the different recombinant CYP6P9a and CYP6P9b proteins exhibit variations in O-dealkylation. Highest activities were observed with diethoxyfluorescein (DEF) and 7-ethoxy-4-trifluoromethylcuomarin (7-EFC) and low activities with 7-ethoxyresorufin (7-ER) and 7-methoxyfluoromethylcuomarin (MFC) (Fig 4A). Higher activities were consistently recorded for CYP6P9b than CYP6P9a, for example in Uganda a 17.9- and 15.9-fold activity was observed respectively toward 7-EFC and DEF for UGANCYP6P9b than UGANCYP6P9a. A comparison of the activity between recombinant proteins shows that for both genes, more evidently for *CYP6P9b*, the recombinant protein from FANG presented a significantly lower activity than proteins from all the field resistant alleles. For example, a 2.85- and 11.3-fold more activity was observed toward DEF, respectively for southern African CYP6P9a and CYP6P9b compared with corresponding proteins from FANG reflecting the higher O-dealkylation property of the resistant alleles. For both genes, no significant difference is observed between alleles from southern Africa and those from East and West Africa suggesting that the nature of the many amino acid changes observed between these alleles and their location do not critically affect their dealkylation activity.

The dealkylation of DEF follows Michaelis-Menten fashion (Fig 4B and 4C respectively), with proteins from resistant alleles exhibiting high activity and affinity compared with those from susceptible FANG (S6 Table). For CYP6P9a, turnover of DEF with MALCYP6P9a was 2-fold higher than obtained with FANGCYP6P9a, leading to catalytic efficiency more than 5-fold higher (p<0.05) (S8A Fig). The catalytic efficiency with UGANCYP6P9a and BENCYP6P9a were also respectively 2- and 6-folds higher than values obtained from FANGCYP6P9a (p<0.05) (S6 Table), reflecting the higher activity in the resistant alleles. For recombinant CYP6P9b, FANGCYP6P9b exhibited lower affinity (*K*_*m*_ roughly two-fold the values obtained with proteins from the resistant alleles), and lower *K*_*cat*_ (S8A Fig and S6 Table). These profound differences in the affinity and maximal activities are reflected in the catalytic efficiencies of CYP6P9b from resistant alleles toward DEF, which were several orders of magnitude (~20 fold) higher than the FANGCYP6P9b activity (p<0.05).

Overall, recombinant proteins from *CYP6P9b* alleles exhibited higher O-dealkylation activity than CYP6P9a alleles with *K*_*cat*_ and catalytic efficiencies on average more than 10-fold the values obtained from CYP6P9a proteins suggesting a higher enzymatic activity in the former compared to the latter.

#### Inhibition assays

To determine whether the different field resistant alleles may exhibit variations in degree of binding to different insecticides, the southern African allele was compared to the Benin allele for *CYP6P9a* and to the Ugandan allele for *CYP6P9b* in an inhibition assay with insecticides from various classes, including Type I pyrethroids (permethrin and deltamethrin), Type II pyrethroids (deltamethrin, λ-cyhalothrin and cypermethrin), the pseudo-pyrethroid etofenprox, organochlorine (DDT), an organophosphate (chlorpyrifos), and two carbamate insecticides (bendiocarb and propoxur). Overall, the alleles exhibited a similar binding pattern to all the insecticides (inhibitors) screened (S8B Fig). Type II pyrethroids showed the most potent inhibitory activity against CYP6P9a*/*CYP6P9b-mediated dealkylation of DEF, with IC_50_s of less than 0.5μM indicating tighter binding compared with Type I pyrethroids, the pseudo-pyrethroid etofenprox and the organophosphate, chlorpyrifos. With DDT, the IC_50_ was averagely 10μM for all proteins excepting UGANCYP6P9b with a much higher value, suggestive of moderate binding of these P450s to this organochlorine, though the low affinity of DDT might be caused by its feeble solubility (low partitioning in water) compared with pyrethroids [29]. IC_50_ values higher than 25μM were obtained with bendiocarb and propoxur suggesting that these insecticides bind poorly to the P450s compared to other inhibitors tested. Tight-binding inhibitors have been defined as compounds with IC_50_ values of <10μM [30].

To establish the robustness of the docking analyses performed with the GOLD software, log IC_50_s of the pyrethroids permethrin, deltamethrin and the pseudo-pyrethroid etofenprox from assays with MALCYP6P9b were compared with the ChemScore values obtained from *in silico* docking of the same insecticides into MALCYP6P9b model. For all three pyrethroids, significantly good correlation was obtained between the experimental IC_50_s and the predicted ChemScores (S8C Fig).

### Assessment of the importance of allelic variation in pyrethroid resistance using transgenic expression in *Drosophila melanogaster*

The extent of the role played by allelic variation of these P450s in pyrethroid resistance was determined in order to establish whether it is alone sufficient to confer resistance *in vivo*, even more than gene over-expression. For this purpose, the most resistant (MAL) and the susceptible alleles (FANG) of each gene were over-expressed *in vivo* through a transgenic expression using the GAL4/UAS system, and their ability to confer resistance compared using contact bioassays with permethrin and deltamethrin. qRT-PCR confirmed that both *CYP6P9a* and *CYP6P9b* were expressed only in the transgenic F_1_ progenies from the crosses between the Actin5C-GAL4 driver line and the different UAS-CYP6P9 lines, and not expressed in the control flies (S9A and S9B Fig) (see S1 Text). Control flies are generated by crossing flies with the same background as the experimental group (but devoid of the UAS element and the candidate gene), with the driver (Actin5C-GAL4) lines to generate Actin5C-GAL4-*null* lines without candidate P450s.

#### Pyrethroids contact bioassays

**Fig 5:**
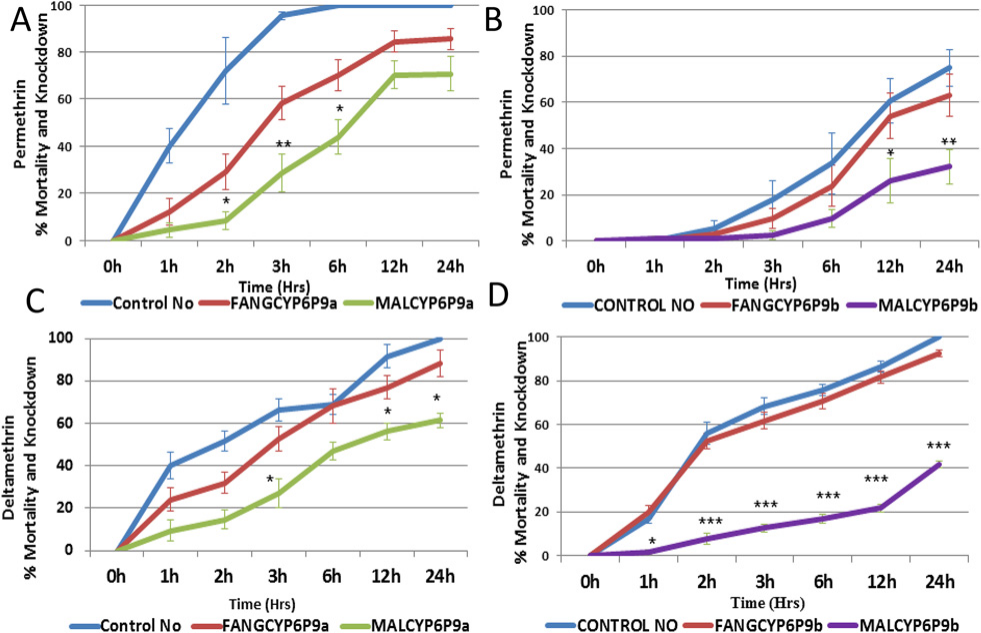
Bioassay results with transgenic flies. (A) Progenies of crosses between Actin5C-GAL4 and UAS-CYP6P9a (transgenic flies over-expressing *CYP6P9a*) with permethrin; (B) transgenic flies over-expressing *CYP6P9b* with permethrin; (C) transgenic flies over-expressing *CYP6P9a* with deltamethrin; and (D) transgenic flies over-expressing *CYP6P9b* with deltamethrin. Data shown as mean ±S.E.M. significantly different: * p<0.05, ** p<0.01 and *** p<0.001.

Bioassays with permethrin and deltamethrin revealed that the flies over-expressing the resistant *MALCYP6P9a* and *MALCYP6P9b* alleles were resistant to pyrethroids, with a very low mortality and knockdown compared with the flies over-expressing the susceptible *FANGCYP6P9a* and *FANGCYP6P9b* alleles, or the control group. For permethrin, progenies of crosses between the Actin5C-GAL4 and UAS-*MALCYP6P9a* (transgenic flies over-expressing *MALCYP6P9a*) showed significantly lower mortality (less than 10%, <30% and <45% in the first 2 hrs, 3 hrs and 6 hrs, respectively) compared with progenies of crosses between Actin5C-GAL4 and UAS-*FANGCYP6P9a* (30%, ~60% and ~70% mortality respectively in 2 hrs, 3 hrs and 6 hrs) (Fig 5). The same pattern was observed for *CYP6P9b* with transgenic flies over-expressing *MALCYP6P9b* showing significantly lower mortalities compared with the transgenic flies over-expressing the susceptible allele (*FANGCYP6P9b*) at the two exposure times of 12 hrs (<30% vs 55%) and 24 hrs (~30% vs ~60%).

With deltamethrin, transgenic flies over-expressing *MALCYP6P9a* showed lower mortalities compared with transgenic flies over-expressing susceptible allele (*FANGCYP6P9a*), although these mortalities were only significant at the third (<30% vs 52%), twelfth and twenty fourth hours of exposure (Fig 5). For *CYP6P9b*, transgenic flies over-expressing the resistant allele *MALCYP6P9b* consistently showed significantly lower mortalities than flies over-expressing susceptible allele (*FANGCYP6P9b*). Statistically significant differences were obtained at every hour of exposure: 1 hr (1.5% vs 20%), 2 hrs (7.5% vs 53%), 3 hrs (12.5% vs 62%), 6 hrs (16% vs 70%), 12 hrs (21% vs 81%) and 24 hrs (41% vs 93%).

Similar to results from *in vitro* assays, for both pyrethroids especially deltamethrin, flies over-expressing *MALCYP6P9b* were significantly more resistant than those over-expressing *MALCYP6P9a* reflecting the pre-eminence of CYP6P9b with respective to pyrethroids metabolism and conferring of resistance.

### Detection of causative mutations using site-directed mutagenesis

#### Expression patterns of CYP6P9b mutant recombinant proteins

Mutant recombinant proteins for *CYP6P9b* were successfully expressed along with the wild type MALCYP6P9b at 21°C and 150 rpm using *E*. *coli* cells *JM109* (see *S1 Text*).

#### Pyrethroid metabolism assays

**Fig 6:**
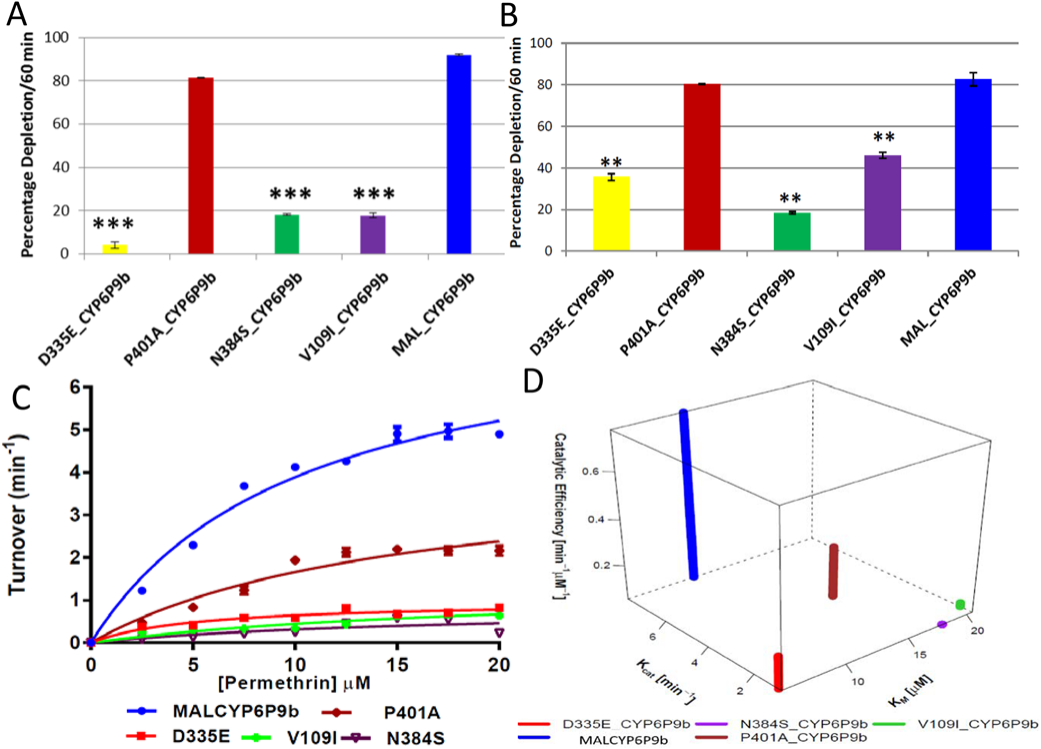
Substrate depletion and kinetic profiles of mutant CYP6P9b recombinant proteins. (A and B) are percentages of depletion of 20μM deltamethrin and permethrin by CYP6P9b mutants, respectively. Results are average of three replicates (n = 3) compared with negative control (-NADPH). (C) Michaelis-Menten plots of mutant CYP6P9b proteins metabolism of permethrin; (D) 4D plot of the kinetic constants and catalytic efficiencies of mutant CYP6P9b proteins for metabolism of permethrin.

Initial depletion assays with permethrin and deltamethrin consistently revealed striking differences in the metabolic profiles of wild type resistant MALCYP6P9b (*wr*MALCYP6P9b) compared with membranes expressing the mutant CYP6P9b proteins. The mutants Asp^335^Glu and Asn^384^Ser, as well as Val^109^Ile exhibited significantly low activity against these pyrethroid insecticides, especially deltamethrin (Fig 6A). Indeed, a 23-, 5.1 and 5.0-fold less depletion is observed for deltamethrin when the resistant allele was mutated to the susceptible allele in positions 335, 109 and 384, respectively. The Pro^401^Ala replacement did not induce a significant change in deltamethrin depletion compared to *wr*MALCYP6P9b. The mutations showed a similar trend against permethrin (Fig 6B) but for Asp^335^Glu and Val^109^Ile a lower impact was observed than against deltamethrin with reduced depletion of 2.15- and 2-fold respectively, compared with amount of permethrin depleted by the *wr*MALCYP6P9b protein. However, for Asn^384^Ser mutant activities toward both permethrin and deltamethrin were similar suggesting that the 384 replacement confer the same impact on both permethrin and deltamethrin.

Metabolism of permethrin by the mutant membranes follow Michaelis-Menten mechanism (Fig 6C) with *wr*MALCYP6P9b having the highest catalytic activity (*K*_*cat*_), values 8-fold than that of Asp^335^Glu, 9-fold the *K*_*cat*_ from Asn^384^Ser and 6-fold as obtained with Val^109^Ile mutant) (S7 Table). Val^109^Ile and Asn^384^Ser mutants exhibited the lowest affinity toward permethrin, with a higher *K*_*m*_, on average 2-fold as obtained with *wr*MALCYP6P9b and 4-fold the *K*_*m*_ of Asp^335^Glu mutant. Pro^401^Ala mutant also exhibited lower *K*_*cat*_, half the value obtained with *wr*MALCYP6P9b but with *K*_*m*_ values on average one-third higher than the wild type enzyme. Thus, the catalytic efficiency *wr*MALCYP6P9b was calculated on average as 3-fold the efficiency of Pro^401^Ala mutant, 4-fold the value obtained with Asp^335^Glu mutant, 13-fold higher than that of Val^109^Ile mutant and 20-fold higher than the efficiency of Asn^384^Ser mutant, showing that each of these amino acid change significantly affect the efficiency of CYP6P9b to metabolise permethrin efficiently.

The differences in the metabolic profiles of the *wr*MALCYP6P9b compared to the mutants can also be rationalised from the spatial positioning of the two residues Asp^335^ and Asn^384^ in MALCYP6P9b model compared with the corresponding residues Glu^335^ and Ser^384^ in FANGCYP6P9b. Overlay of MALCYP6P9b and FANGCYP6P9b models revealed striking differences in the overall 3D folding and backbone of the proteins (S10 Fig). These differences may result in differences in the manner through which these residues interact with the substrates or the redox partners with impact on catalysis (see *S1 Text*).

#### Fluorogenic probes assays

Differences in activities toward probe substrates were also observed between MALCYP6P9b and the mutants (S11A Fig). The wild type membrane exhibited higher activity towards diethoxyfluorescein (DEF) with catalytic efficiencies more than 8-fold, 10-fold and 15-fold greater than values from Asp^335^Glu, Val^109^Ile and Asn^384^Ser mutants, respectively (see *S1 Text* for further details).

## Discussion

Detecting insecticide resistance at early stage is one of the prerequisite for the design and implementation of effective insecticide resistance management strategies. DNA-based diagnostic tools are essential for this purpose, but the diagnostics require a thorough understanding of the molecular basis of insecticide resistance. In this study, we dissected the molecular basis of a monooxygenase-mediated resistance to pyrethroid in one of the major African malaria vectors *An*. *funestus* demonstrating that (i) allelic variation in cytochrome P450 genes is a key molecular mechanism conferring pyrethroid resistance in field mosquitoes, and (ii) detecting key amino acid changes in resistant P450 alleles responsible for pyrethroids-metabolising efficiency. The finding of these resistance markers could help in the design of DNA-based diagnostic tools that will facilitate the detection and tracking of such resistance markers in the field.

### Allelic variation is a key mechanism conferring pyrethroid resistance

This study has provided several evidences supporting the key role of allelic variation in conferring pyrethroid resistance.

#### Evidences from Africa-wide patterns of genetic variability of CYP6P9a and CYP6P9b alleles and in silico prediction of activity

Analysis of nucleotide sequences of different alleles of *CYP6P9a* and *CYP6P9b* across Africa revealed that the alleles from resistant populations are undergoing directional selection, with reduced genetic diversity and beneficial mutations selected, compared with the alleles from the susceptible population, FANG. Such signature of selection is similar to that reported in *CYP6G1* P450 from *D*. *melanogaster* [31]. *In silico* prediction suggested that the allelic variants differ in their activity towards pyrethroids. Pyrethroids docked into the models of susceptible alleles in unproductive mode or away from the heme catalytic centre for catalysis to occur. *In silico* predictions have been applied in several studies to predict the ability of insect P450s to metabolise pyrethroids and other substrates [16, 19, 22].

#### Evidences from variation in metabolic activity towards pyrethroid and probe substrates

Metabolism assays established that the resistant alleles of *CYP6P9a* and *CYP6P9b* metabolise pyrethroid insecticides with higher turnover compared to the susceptible alleles. Highest activities were obtained from southern African alleles consistent with the highest pyrethroid resistance recorded in this region [4, 17, 32]. Kinetic parameters also differ significantly between resistant alleles of *CYP6P9a* and *CYP6P9b* and the susceptible ones. The resistant alleles exhibited high turnover (high *K*_*cat*_) and high affinity (lower *K*_*m*_) compared with the susceptible alleles, which translates into a very high efficiency of pyrethroid metabolism several-fold in the former compared with the latter. These findings established that allelic variation is impacting the pyrethroid metabolising efficiencies of resistant alleles of *CYP6P9a* and *CYP6P9b*. The kinetic parameters are compared with results established from several other studies with pyrethroids, as well as the probes assays in more detail in *S1 Text.*

#### Evidence from transgenic expression

Expression of alleles of *CYP6P9a* and *CYP6P9b* from resistant populations alone confers resistance to pyrethroids permethrin and deltamethrin *in vivo*, showing that allelic variation could even be more important in conferring resistance than gene over-expression. However, because resistant *CYP6P9a* and *CYP6P9b* alleles are always found to be also over-expressed in the field, it is likely that allelic variation combines with increased expression in this resistance mechanism as previously reported in other species notably in humans [33]. The ability of the transgenic expression to reveal phenotypic difference caused by gene polymorphism in *CYP6P9a* and *CYP6P9b* further highlights the reliability of this approach in characterising the functional role of genes in relation to phenotypes as done in other species such as *Tribolium castaneum* (*CYP6BQ9*) [34] and *D*. *melanogaster* (*CYP6G1 and CYP12D1*) [35].

Impact of allelic variation has previously been reported in the *CYP6A2* P450 gene in *D*. *melanogaster* with regard to DDT resistance [36] suggesting that allelic variation of key resistance genes could be common in insect species (see *S1 Text* for further details).

### Key amino acid changes control pyrethroid resistance and could lead to DNA-based diagnostic tools

Detection of DNA-based markers of metabolic resistance has so far proved challenging because of the redundancy of genes involved but also because of the multitude of mechanisms through which these mechanisms can operate. Here, using site-directed mutagenesis and comparative recombinant enzyme characterisation we established, for the first time in our knowledge in a mosquito species, that three amino acid replacements in the P450 *CYP6P9b* are responsible for high metabolic activity toward pyrethroids as their replacement in *MALCYP6P9b* with variants from the susceptible *FANGCYP6P9b* correlated with significant loss of catalytic activity and metabolic efficiency towards permethrin. We suggest that the three residues (Val^109^, Asn^384^ and Asp^335^) in the protein from the resistant alleles of *CYP6P9b* contribute in unique ways to create ensemble complementarity groups on the P450 active sites, a potent pharmacophore which recognise key features on the pyrethroid substrates, allowing optimal inter-molecular interactions and enhancing affinity and/or catalysis.

Impact of amino acid changes in metabolic activity of CYP450 genes have been described previously in relation to metabolisms of other chemicals but not against pyrethroids. Indeed, the Glu^318^Asp substitution in human *CYP1A2* has been shown to increase the *K*_cat_ of O-dealkylation of 7-ethoxycoumarin 13-fold [37] (see S1 Text).

As these are the first markers of metabolic resistance to pyrethroids involving P450s in *An*. *funestus* and/or even other mosquito species, these mutations should facilitate the design of DNA-based diagnostic tests for tracking resistance in the field. However, unlike the case of DDT-resistance gene *GSTe2* (Leu^119^Phe substitution) in *An*. *funestus*, the *CYP6P9a* and *CYP6P9b* are duplicated genes with high sequence similarity which will require particular attention in the design of diagnostic assays. The detection of these causative mutations in *CYP6P9b* also set a pace for studies of this kind to be carried out in other insect vectors for which allelic variation in key resistance genes could also play an important role.

### Conclusion

This study presents a detailed dissection of the genetic and molecular basis of metabolic resistance to insecticide in a major malaria vector demonstrating that allelic variation of key P450 genes is responsible for the resistance to pyrethroids, the bed nets insecticides. Key amino acid changes between resistant and susceptible alleles specifically, three residues, Val^109^, Asp^335^ and Asn^384^ in the resistant *CYP6P9b* alleles accounted for these metabolic differences. The finding and characterisation of these resistance markers paves a way to design a DNA-based diagnostic test that can allow tracking of these resistant alleles across Africa, enabling the design of evidenced-based resistance management strategies to mitigate the impact of this resistance on the success of ongoing and future control interventions.

## Methods

### Sequence characterisation and *in silico* analysis of *CYP6P9a* and *CYP6P9b* alleles

#### Polymorphism analysis of CYP6P9a and CYP6P9b alleles

To establish the presence of allelic variation in both *CYP6P9a* and *CYP6P9b*, the full-length cDNA of both genes was amplified from permethrin resistant mosquitoes from three regions of Africa with different resistance profiles, as well as from the susceptible laboratory strain FANG, originally from Angola. The resistant populations were *An*. *funestus s*.*s*. from southern Africa: Chikwawa in Malawi, Chokwe in Mozambique and Katete in Zambia [4, 17, 32], East-Africa: Tororo in Uganda [38], and West-Africa: Pahou in Benin [39]. Pyrethroid-resistance profiles of these mosquito populations as well as their relevant transcriptional analysis have been previously published [17, 38, 39]. The fully susceptible FANG from Calueque, southern Angola [40] was utilised as a susceptible population.

Full-length sequences of *CYP6P9a* and *CYP6P9b* were amplified using cDNA synthesised from total RNA extracted from three batches of 10 mosquitoes from Benin, Uganda, Malawi, Mozambique and Zambia, and the FANG, as previously described [17] (primers given in S8 Table). Polymorphisms were detected through manual examination of sequence traces using BioEdit version 7.2.3.0 [41] and sequence differences in multiple alignments using CLC Sequence Viewer 6.9 (http://www.clcbio.com/). Different haplotypes were compared by constructing a phylogenetic maximum likelihood tree using MEGA 6.0 [42]. Genetic parameters of polymorphism of both genes for each sample were determined using DnaSP 5.10 [43]. Locations of amino acids differences within the sequences of *CYP6P9a* and *CYP6P9b* were mapped by identifying helices A-L and substrate recognition sites (SRS1-6) using crystal structures [44, 45] and structurally conserved regions using *CYPED* database [46].

#### Comparison of various CYP6P9a and CYP6P9b alleles using molecular docking simulation

To predict the impact of observed amino acid changes on the structure of *CYP6P9a* and *CYP6P9b* models of both genes were created using MODELLER *9v2* [47] and CYP3A4 (PDB: 1TQN) [48] as a template, with 33% and 32% identity, respectively for CYP6P9a and CYP6P9b sequences. Ligand structures were retrieved from ZINC^12^ library (https://zinc.docking.org/). 3D protein models and ligands were prepared for docking using Molegro Molecular Viewer *2*.*5* (http://www.clcbio.com/). To predict the pattern of interaction between the genes and insecticides, docking was carried out with GOLD 5*v2* [49], with ChemScore scoring function [50] and active site defined as a cavity of 20Å radius centred above the heme iron. 50 binding poses were obtained for each ligand (permethrin, deltamethrin and etofenprox) and scored according to binding parameters and the conformation of ligands in the active site of the respective P450s. Figures were prepared using the PyMOL 1.7 [51].

#### *In silico* prediction of substrate access/product egress channels

To identify potential substrate access and/or product egress channels between the binding sites of pyrethroids to the bulk solvent, comparative channels search was conducted using models generated from sequences of resistant alleles and those from the susceptible (FANG) alleles. Channel searches was conducted using the algorithm tool CAVER 3.1 [52], with settlings for tunnels calculations as described in the PyMOL plugin [53].

### *In vitro* functional characterisation of metabolic activity of various recombinant CYP6P9a and CYP6P9b proteins

#### Cloning and heterologous expression of recombinant CYP6P9a and CYP6P9b in *E*. *coli*

Recombinant enzymes of both *CYP6P9a* and *CYP6P9b* genes were expressed for the different alleles. Expression plasmids pB13::ompA+2-*CYP6P9a* and pB13::ompA+2-*CYP6P9b* for the alleles: Benin-*CYP6P9a* and–*b* (hereby after *BENCYP6P9a* and *BENCYP6P9b*), Uganda (*UGANCYP6P9a* and *UGANCYP6P9b*), Malawi (*MALCYP6P9a* and *MALCYP6P9b*) and FANG (*FANGCYP6P9a* and *FANGCYP6P9b*) were constructed by fusing cDNA fragment from a bacterial ompA+2 leader sequence with its downstream ala-pro linker to the NH_2_-terminus of the P450 cDNA, in frame with the P450 initiation codon, as described [54]; and then cloned into *Nde*I*-* and *Xba*I-linearised pCW-ori+ vector [55]. Details of PCR conditions used to create this type of expression plasmid cassettes have already been described [4, 17] and list of primers are provided in S8 Table.

For all alleles, *E*. *coli JM109* cells were co-transformed with the P450 expression cassettes and a plasmid containing the *An*. *gambiae* cytochrome P450 reductase (pACYC-*AgCPR*) fused to *pelB* leader sequence [56]. Membrane expression and preparations, measurement of P450 content, measurement of cytochrome *c* reductase activity, cytochrome b_5_ expression and measurement of its content were carried out as previously described [19, 57, 58].

#### Comparative assessment of pyrethroids metabolic activity of various recombinant *CYP6P9a* and *CYP69b* alleles using metabolism assays

To establish whether allelic variation in *CYP6P9a* and *CYP6P9b* impact their metabolic activities toward insecticides, pyrethroids were screened with the recombinant enzymes from these genes. As previously described [17, 28], 0.2M Tris-HCl and NADPH-regeneration components (1mM glucose-6-phosphate, 0.25mM MgCl_2_, 0.1mM NADP and 1U/ml glucose-6-phosphate dehydrogenase) were added to the bottom of 1.5ml tube chilled on ice. Membrane expressing recombinant CYP6P9a/-b and *Ag*CPR, and reconstituted cytochrome b_5_ were added to the side of the tube and pre-incubated for 5 minutes at 30°C, with shaking at 1,200 rpm to activate the membrane. 20μM of test insecticide was then added into the final volume of 0.2ml (~2.5% v/v methanol), and reaction started by vortexing at 1,200 rpm and 30°C for 1 hour. Reactions were quenched with 0.1ml ice-cold methanol and incubated for 5 more minutes. Tubes were then centrifuged at 16,000 rpm and 4°C for 15 minutes, and 150μl of supernatant transferred into HPLC vials for analysis. Reactions were carried out in triplicates with experimental samples (+NADPH) and negative controls (-NADPH). 100μl of sample was loaded onto an isocratic mobile phase (90:10 v/v methanol to water) with a flow rate of 1ml/min, monitoring wavelength of 226nm and peaks separated with a 250mm C18 column (Acclaim 120, Dionex) on Agilent 1260 Infinity at 23°C. Enzyme activity was calculated as percentage depletion (the difference in the amount of insecticide(s) remaining in the +NADPH tubes compared with the–NADPH) and a t-test used for statistical analysis.

Steady-state kinetic parameters for each allele were obtained with permethrin and deltamethrin by measuring the rate of reaction under linear conditions for 10 minutes while varying the substrates concentrations (2.5μM-20μM). Reactions were performed in triplicates both for +NADPH (experimental tubes) and–NADPH (negative control). *K*_*m*_ and *V*_*max*_ were established from the plot of substrate concentrations against the initial velocities and fitting of the data to the Michaelis-Menten module using the least squares non-linear regression, as described in the GraphPad Prism 6.03 Software (GraphPad Inc., La Jolla, CA, USA).

#### Comparative assessment of metabolic activities of recombinant *CYP6P9a and CYP6P9b* using fluorescent probe assays

To detect potential differences in substrate preferences, recombinant proteins from the various alleles of CYP6P9a and CYP6P9b were screened with fluorescent probes, kinetic analysis carried out, and inhibition assays conducted. Probes such as diethoxyfluorescein undergo P450-mediated O-dealkylation with large Stokes shift which can be measured fluorometrically [59].

Four probes: 7-ethoxyresurofin (7-ER), 7-ethoxy-4-trifluoromethylcoumarin (7-EFC), 7-methoxy-4-trifluoromethylcoumarin (MFC) and diethoxyfluorescein (DEF) were initially tested with the various recombinant CYP6P9a and CYP6P9b proteins. In a total volume of 225μl containing ~10pmol P450, buffered with 50mM potassium phosphate buffer (KPi at pH 7.4 with 5mM MgCl_2_), 1μM probe substrate was added. Membranes were activated for 5 minutes at 37°C in fluorescence spectrophotometer Infinite M200 (TECAN) before 25μl NADPH regeneration buffer (1mM glucose-6-phosphate (G6P), 0.25mM MgCl_2_, 0.1mM NADP and 1U/ml glucose-6-phosphate dehydrogenase (G6PDH) prepared in KPi pH 7.4) was added. All reactions were conducted in three replicates with negative control (–NADPH) devoid of the regeneration buffer. Rate of fluorescent product formation for 7-ER (ʎ_exc_ = 544nm, ʎ_emi_ = 590nm), 7-EFC and MFC (ʎ_exc_ = 410nm, ʎ_emi_ = 535nm) and DEF (ʎ_exc_ = 485nm, ʎ_emi_ = 530nm) was determined by linear regression of measurement between 2 and 10 minutes after start of the reaction. Results were analysed using Magellan v6.2 software.

For kinetics, 0 to 2μM diethoxyfluorescein was assayed with amount of membranes which produced optimal activities (10pmol for CYP6P9a and 3.33pmol for CYP6P9b) in a total volume of 250μl. The protocol was as outlined above, only that the substrate concentration varies and incubation was done under conditions established to be linear with respect to time. Steady-state kinetic parameters were obtained by measuring the rate of reaction for 10 minutes while varying the substrate concentration from 0 to 2μM. *K*_*m*_ and *V*_*max*_ were established from the plot of substrate concentrations against the initial velocities through a non-linear regression, by fitting the data to the Michaelis-Menten equation using GraphPad Prism 6.03 (GraphPad Software).

To further assess differences in the degree of binding of the various recombinant proteins to insecticides from different classes, inhibition assay was conducted with DEF and ten insecticides (inhibitors). The potent P450s inhibitor miconazole [60] was utilised as a positive control inhibitor. IC_50_ determination was conducted as described in previous studies [21, 61]. In a total volume of 225μl buffered with 50mM KPi (pH 7.4), containing 0.1–0.13μM DEF (~*K*_*m*_ values), 10pmol CYP6P9a or 3.33pmol CYP6P9b, 5μl (25μM) test inhibitors or miconazole was spiked in the top wells and serially diluted into eight-fold concentrations (25–0.011μM). The 96 well-plate was pre-incubated for 5 minutes at 37°C and then 25μl regeneration buffer (7.8mg glucose-6-phosphate (G6P), 0.25mM MgCl_2_, 1.7mg NADP, 6U/ml glucose-6-phosphate dehydrogenase and 2% w/v NaHCO_3_) incorporated. Fluorescence was monitored for 21 cycles at interval of 1 minute with shaking at every step. Results were analysed using Magellan v6.2 software and inhibition at each inhibitor concentration and incubation time calculated as residual control activity towards DEF.

### Comparative transgenic expression of resistant and susceptible alleles of *CYP6P9a* and *CYP6P9b* in *D*. *melanogaster*

To assess whether allelic variation in *CYP6P9a* and *CYP6P9b* was the most important factor conferring pyrethroid resistance even more than gene over-expression, a resistant (Malawi) and a susceptible (FANG) alleles for each gene were independently expressed in *D*. *melanogaster*, using the GAL4-UAS system. The hypothesis being that if sequence variation in these two genes is the key determinant of pyrethroid resistance, then over-expression of resistant alleles of both genes will confer more resistance to pyrethroids than observed by the over-expression of the susceptible alleles.

#### Cloning and construction of transgenic plasmids

Four transgenic lines, UAS-*MALCYP6P9a*, UAS-*FANGCYP6P9a*, UAS-*MALCYP6P9b* and UAS-*FANGCYP6P9b* were constructed and crossed with Actin5C-GAL4 lines to drive ubiquitous expression. Primers used for this PCR are given in S8 Table. PCR conditions, cloning techniques, construction of transgenic flies and expression of candidate genes was conducted as described in previous studies [4], with more details provided in *S1 Text*. For control group, flies with the same background as the UAS transgenic lines but devoid of the UAS construct with the candidate gene were crossed with the driver Actin5C-GAL4 line to generate Actin5C-GAL4-*null* flies.

#### Insecticides contact bioassays

Insecticide (2% permethrin and 0.15% deltamethrin-impregnated) filter papers were prepared in acetone and Dow Corning 556 Silicone Fluid (BDH/Merk, Germany) and kept at 4°C prior to bioassay. These papers were then rolled and introduced into 45 cc plastic vials. The vials were then plugged with cotton wool soaked in 5% sucrose. 20–25 (2–4 days old, post-eclosion F_1_ females) were selected for the bioassays and introduced into the vials. Mortality plus knockdown was scored after 1 hr, 2 hrs, 3 hrs, 6 hrs, 12 hrs and 24 hrs post-exposure to the discriminating dose of the insecticides. For each assay, at least six replicates were performed for both experimental flies and control, and t-test used to carry out statistical analysis of mortality plus knockdown observed between experimental groups and control.

#### qRT-PCR validation of over-expression of transgenes

To confirm expression of the candidate genes in the experimental flies and its absence in the control groups, qRT-PCR was carried out as described previously [4, 17]. RNA was extracted from three pools of 5 F_1_ experimental and control flies separately and cDNA synthesized as described. qRT-PCR for both *CYP6P9a* and *CYP6P9b* mRNA was conducted using the primers given in S8 Table, with normalization using the housekeeping gene *RPL11*.

### Detection of causative mutations in *CYP6P9a* and *CYP6P9b* using site-directed mutagenesis

To detect the key amino acid changes conferring high pyrethroid-metabolising efficiency in resistant *CYP6P9a* and *CYP6P9b* alleles, a hypothesis-driven approach was used to select candidate amino acids from resistant alleles which were mutated into variants existing in the susceptible alleles, *FANGCYP6P9a* and *FANGCYP6P9b*. The hypothesis being that if the target residue is linked with high pyrethroid metabolising-activity in the resistant allele then a significant loss of activity will be obtained when mutated into a variant from the susceptible allele. For *CYP6P9a* the substitution aimed at were Ser^320^Tyr, Phe^431^Ser, Gln^301^His and a double mutant Leu^63^Phe_Lys^66^Gln. For *CYP6P9b* the following replacements were effected: Val^109^Ile, Asp^335^Glu, Asn^384^Ser and Pro^401^Ala. For both genes, the first amino acid in each case represents residue from the resistant allele and the second amino acids being variants present in the susceptible allele. Amplification was achieved using mutagenic primers (S8 Table) in a whole plasmid primer extension PCR followed by self-ligation at transformation step [62, 63]. The PCR was carried out using plasmidic pJET1.2::ompA+2-*MALCYP6P9a* and pJET1.2::ompA+2-*MALCYP6P9b* respectively as templates, with Phusion HotStart II High-Fidelity DNA Polymerase (Thermo SCIENTIFIC, USA). In a reaction mix containing 1X Buffer (with 7.5mM MgCl_2_), 80μM dNTP mixes, 0.3μM each of forward and reverse mutagenic primers, 2-4ng plasmidic DNA template, 0.04U/μl of Phusion HotStart Taq and sterile water was added to give 50μl. The reaction was started by preheating the mixture to 98°C for 10 minutes; and then followed by 35 cycles each of 94°C for 30 seconds; annealing (at 65°C) for 30 seconds and extension at 72°C for 2.5 minutes. This is then followed with final extension at 72°C for 10 minutes, and 4°C (hold). The PCR product was incubated for 1 hr with 2μl of 1X FastDigest Buffer and 1μl of *Dpn*I (Thermo SCIENTIFIC), at 37°C to digest the *Dam* ^+^-methylated parental template [62]. 4μl of the digest was then transformed into *E*. *coli DH5α*. Positive colonies were mini-prepped and sequenced on both strands to confirm presence of desired mutations. Mutagenic plasmids of *MALCYP6P9b* were successfully digested with *Nde*I and *Xba*I restriction enzymes, gel extracted with QIAquick Gel Extraction Kit (QIAGEN) and ligated into pCWOri+ already linearized with the same restriction enzymes, to construct the mutagenic cassettes pB13::ompA+2-*MALCYP6P9b;* mutagenic plasmids of *MALCYP6P9a* could not be cut out from the pJET1.2 construct and consequently the P450 insert could not be introduced into the expression vector pCWOri+. The mutant plasmids of *MALCYP6P9b* were then co-transformed together with *AgCPR* into *JM109* and functional membranes expressed as described previously. Efforts to introduce mutations into the constructs pB13::ompA+2-*CYP6P9a* and pB13::ompA+2-*CYP6P9b* directly were not successful due possibly to the larger size of pCWori+ plasmid and as such amplification of mutants from *MALCYP6P9a* plasmids were not successful.

Recombinant MALCYP6P9b membranes expressed from mutant plasmids were used alongside the wild type recombinant MALCYP6P9b (*wr*MALCYP6P9b) to screen for metabolic activities against Type I and Type II pyrethroids (permethrin and deltamethrin, respectively), in order to determine the functional impact of each amino acid changed on metabolism of insecticides. In addition, fluorescent probes were also screened to establish differences in the O-dealkylation activity of the mutants compared with the wild type enzyme. All *in vitro* and kinetic analyses were conducted as described above. However, as different mutants expressed with varying concentrations all assays were conducted within linear range, with the amount of enzyme and time which produced highest activities.

### Accession numbers

The DNA sequences reported in this paper have been deposited in the GenBank database (accession numbers: GenBank KR866022-KR866069).

## Supporting Information

S1 Fig Comparison of CYP6P9a amino acid sequences from FANG allele and resistant alleles. Red, solid boxes represent helices A-L; blue, dashed boxes represent the predicted substrate recognition sites 1–6 (SRS1-6). Amino acid substitutions are highlighted in pink.
(TIF)

S2 Fig Structurally-conserved regions and motifs of *An*. *funestus* CYP6P9b alleles from resistant individuals and susceptible FANG. Red, solid boxes represent motifs and blue, solid boxes correspond with the protein *β*-sheets. Amino acids substitutions are highlighted in red.
(TIF)

S3 Fig Predicted binding mode of permethrin in (A) MALCYP6P9a, (B) FANGCYP6P9a, (C) MALCYP6P9b and (D) FANGCYP6P9b models. Permethrin is in stick format and orange. Heme atoms are in stick format and grey. Distance between possible sites of metabolism and heme iron are annotated in Angstrom.
(TIF)

S4 Fig Predicted binding mode of deltamethrin in (A) MALCYP6P9a, (B) FANGCYP6P9ba (C) MALCYP6P9b and (D) FANGCYP6P9b models. Deltamethrin is in stick format and cyan. Heme atoms are in stick format and grey. Distance between possible sites of metabolism and heme iron are annotated in Angstrom.
(TIF)

S5 Fig Trajectory of the substrate access channel *pw2a* in the models of (A) MALCYP6P9b and (B) FANGCYP6P9b docked with deltamethrin. Helix I and BʹC loop are annotated; *pw2a* is in blue colour; deltamethrin is in stick format and pink; heme atoms are in stick format and grey. Key residues Val^109^ and Ile^109^ are highlighted in stick format and yellow colour, and distance to the channel annotated.
(TIF)

S6 Fig (A) Membrane contents and (B) Cytochrome P450 Reductase activity of various recombinant proteins expressed from different *CYP6P9a* and *CYP6P9b* alleles. (TIF)

S7 Fig Michaelis-Menten plot of metabolism of permethrin (A) and (B) and deltamethrin (C) and (D) by various recombinant proteins of *CYP6P9a* and *CYP6P9b* alleles. Each point is a mean ± S.E.M. of three independent replicates.
(TIF)

S8 Fig (A) 4D plot of the kinetic constants and catalytic efficiencies of CYP6P9a and CYP6P9b metabolism of diethoxyflourescein. (B) and (C) Inhibition assays of recombinant CYP6P9a and CYP6P9b with diethoxyfluorescein and insecticide inhibitors. Insecticides tested include Type I (permethrin and bifenthrin) and Type II (deltamethrin, λ-cyhalothrin and cypermethrin) pyrethroids, etofenprox and non-pyrethroid insecticides; (B) Mean IC_50_ of the test insecticide inhibitors against CYP6P9a and CYP6P9b metabolism of diethoxyfluorescein. Data represent mean IC_50_ at eight concentrations of each insecticide plus or minus standard deviation. Error bars represent variation in the values of the IC_50_ between different concentrations. For this assay, miconazole was used a positive control inhibitor; (C) Correlation between the IC_50_ of test insecticides inhibitors on MALCYP6P9b metabolism of diethoxyfluorescein with ChemScore values from docking with GOLD: Numerals I, II and III represents permethrin, deltamethrin and etofenprox, respectively. r^2^ = regression coefficient.
(TIF)

S9 Fig qRT-PCR validation of expression of CYP6P9a and CYP6P9b in transgenic flies: Fold change of *CYP6P9a* (A) and *CYP6P9b* (B) mRNA from progenies of crosses between Actin5C-GAL4 and UAS-*CYP6P9a*/UAS-*CYP6P9b*, expressing transgenic *CYP6P9a* and *CYP6P9b*, relative to house-keeping gene *RPL11*. (TIF)

S10 Fig Overlay of MALCYP6P9b (helices in spectrum and cartoon format) and FANGCYP6P9b (helices in purple and cartoon) models with deltamethrin docked in productive pose in MALCYP6P9b adopted for FANGCYP6P9b. (A) FANGCYP6P9b-Ser^384^: yellow stick; FANGCYP6P9b-Arg^385^: green stick with superscript F; MALCYP6P9b-Asn^384^: blue stick; MALCYP6P9b-Arg^385^: red stick with superscript M; heme atoms: stick format and grey; deltamethrin in stick format and pink; (B) MALCYP6P9b-Asp^335^ is in stick form and blue colour, and FANGCYP6P9b-Glu^335^ is in red colour.
(TIF)

S11 Fig Metabolism of probe substrates by mutant membranes of *CYP6P9b*. (A) O-dealkylation of seven fluorescent probes by the recombinant CYP6P9b mutants. The solid bars indicate average of significant activity in three experimental replicates compared to negative controls (-NADPH). RBE, resorufin benzylether; 7-ER, 7-ethoxyresorufin; RME, resorufin methylether; RPE, resorufin pentylether; DEF, diethoxyfluorescein; 7-EFC, 7-ethoxy-4-trifluoromethylcuomarin; MFC, 7-methoxy-4-trifluoromethylcuomarin. (B) Michaelis-Menten plots of mutant CYP6P9b proteins metabolism of diethoxyfluorescein; (C) 4D plot of the kinetic constants and catalytic efficiencies of mutant CYP6P9b proteins’ metabolism of diethoxyfluorescein.
(TIF)

S1 Table Summary statistics for polymorphism of *CYP6P9a* and *CYP6P9b* haplotypes across Africa. (DOCX)

S2 Table Key nucleotide polymorphisms and amino acid substitutions between sequences of *CYP6P9a* and *CYP6P9b* from resistant alleles compared with FANG. (DOCX)

S3 Table Binding parameters for permethrin and deltamethrin docked to the active sites of CYP6P9a and CYP6P9b models. (DOCX)

S4 Table Percentage depletion of pyrethroid insecticides by various recombinant CYP6P9a and CYP6P9b proteins. (DOCX)

S5 Table Kinetic constants for metabolism of permethrin and deltamethrin by various recombinant proteins of CYP6P9a and CYP6P9b. (DOCX)

S6 Table Kinetic constants for recombinant CYP6P9a and CYP6P9b metabolism of diethoxyfluorescein. (DOCX)

S7 Table Kinetic constants for recombinant mutants of CYP6P9b metabolism of permethrin and diethoxyfluorescein. (DOCX)

S8 Table List of primers used in this study. (DOCX)

S1 Text (DOCX)
