# Supplementary figures and images for: Allelic Variation of Cytochrome P450s Drives Resistance to Bednet Insecticides in a Major Malaria Vector
Source: PLoS Genet. 2015 Oct 30;11(10):e1005618. doi: 10.1371/journal.pgen.1005618 (PMC4627800; doi:10.1371/journal.pgen.1005618)

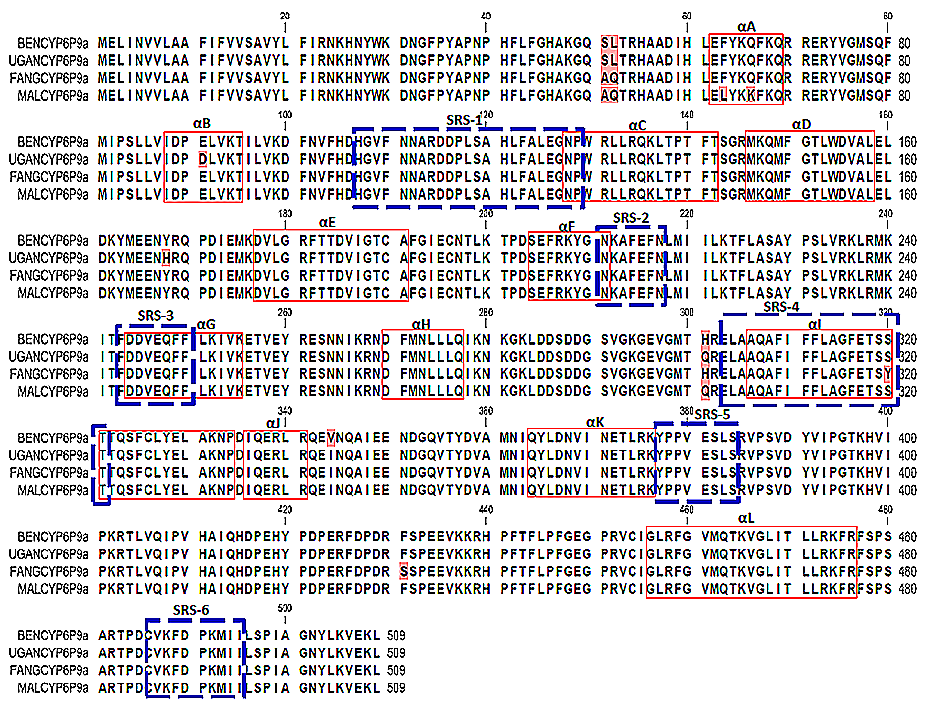

Supplement: S1 Fig — Red, solid boxes represent helices A-L; blue, dashed boxes represent the predicted substrate recognition sites 1–6 (SRS1-6). Amino acid substitutions are highlighted in pink. (TIF) [file pgen.1005618.s001.tif]

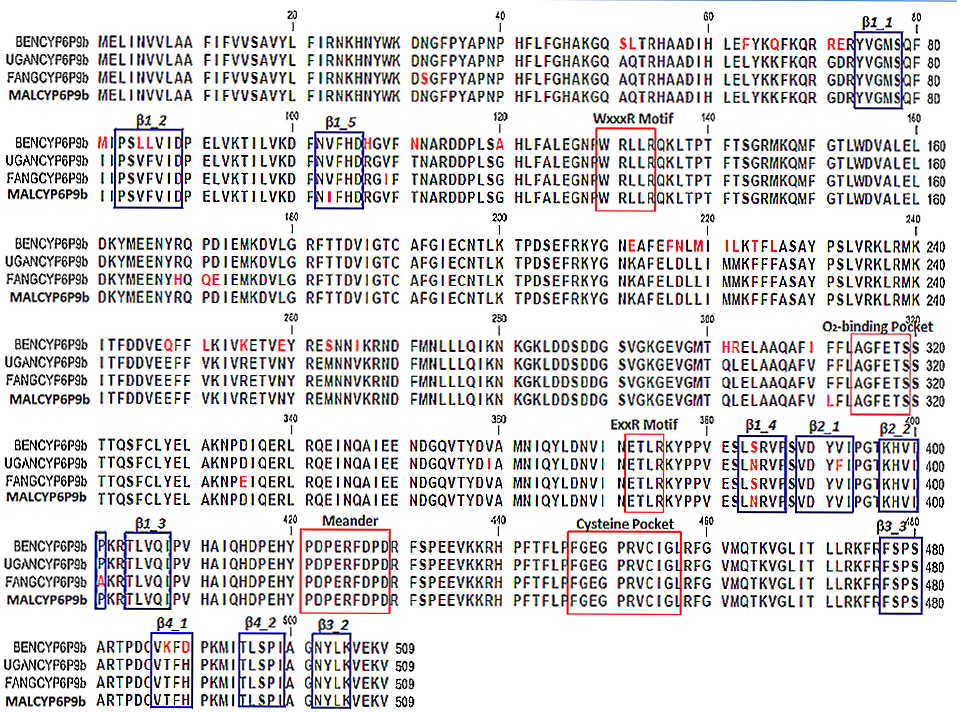

Supplement: S2 Fig — Red, solid boxes represent motifs and blue, solid boxes correspond with the protein β-sheets. Amino acids substitutions are highlighted in red. (TIF) [file pgen.1005618.s002.tif]

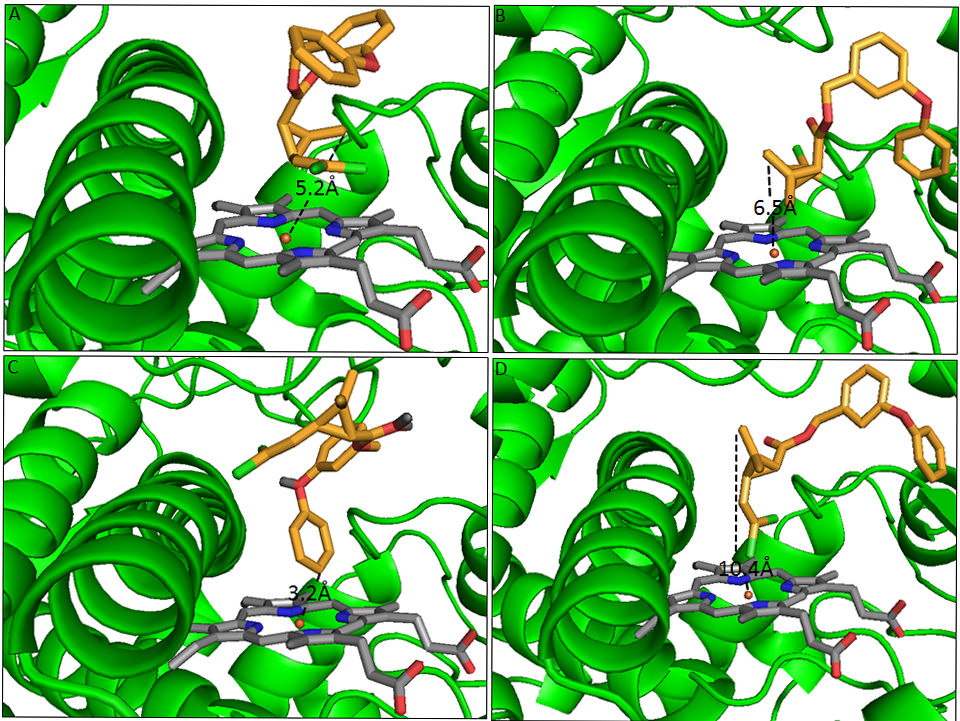

Supplement: S3 Fig — Permethrin is in stick format and orange. Heme atoms are in stick format and grey. Distance between possible sites of metabolism and heme iron are annotated in Angstrom. (TIF) [file pgen.1005618.s003.tif]

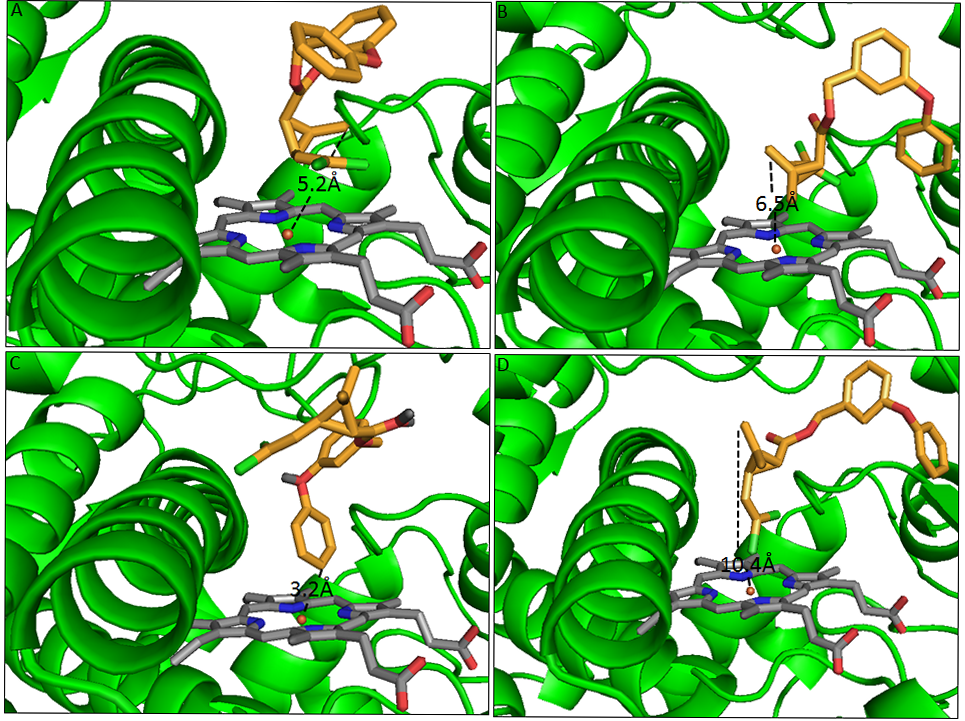

Supplement: S4 Fig — Deltamethrin is in stick format and cyan. Heme atoms are in stick format and grey. Distance between possible sites of metabolism and heme iron are annotated in Angstrom. (TIF) [file pgen.1005618.s004.tif]

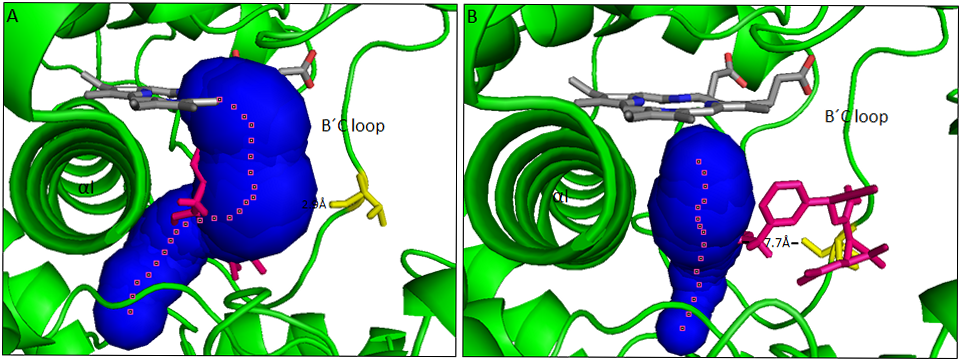

Supplement: S5 Fig — Helix I and BʹC loop are annotated; pw2a is in blue colour; deltamethrin is in stick format and pink; heme atoms are in stick format and grey. Key residues Val109 and Ile109 are highlighted in stick format and yellow colour, and distance to the channel annotated. (TIF) [file pgen.1005618.s005.tif]

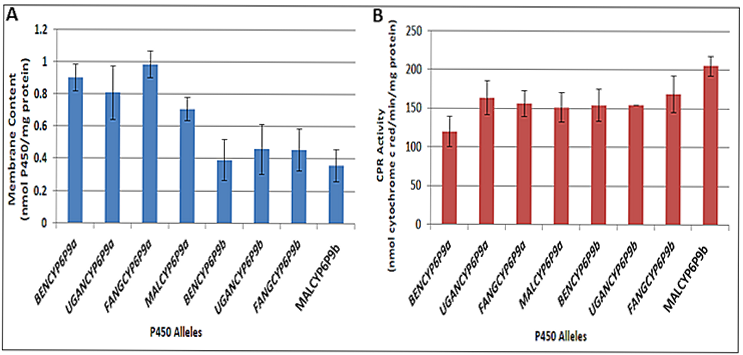

Supplement: S6 Fig — (TIF) [file pgen.1005618.s006.tif]

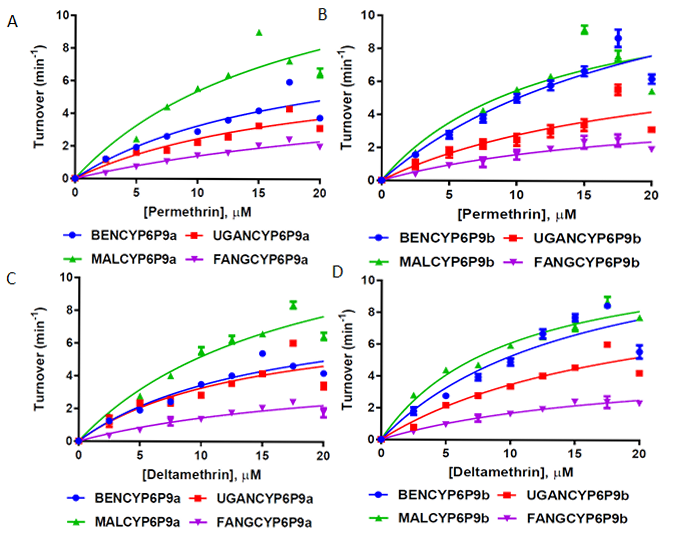

Supplement: S7 Fig — Each point is a mean ± S.E.M. of three independent replicates. (TIF) [file pgen.1005618.s007.tif]

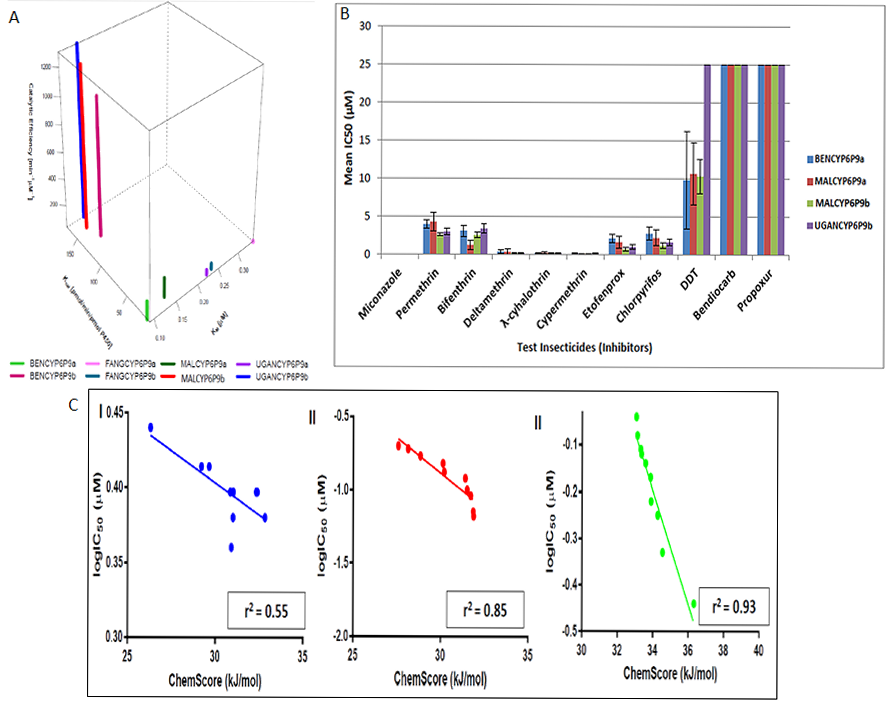

Supplement: S8 Fig — Insecticides tested include Type I (permethrin and bifenthrin) and Type II (deltamethrin, λ-cyhalothrin and cypermethrin) pyrethroids, etofenprox and non-pyrethroid insecticides; (B) Mean IC50 of the test insecticide inhibitors against CYP6P9a and CYP6P9b metabolism of diethoxyfluorescein. Data represent mean IC50 at eight concentrations of each insecticide plus or minus standard deviation. Error bars represent variation in the values of the IC50 between different concentrations. For this assay, miconazole was used a positive control inhibitor; (C) Correlation between the IC50 of test insecticides inhibitors on MALCYP6P9b metabolism of diethoxyfluorescein with ChemScore values from docking with GOLD: Numerals I, II and III represents permethrin, deltamethrin and etofenprox, respectively. r2 = regression coefficient. (TIF) [file pgen.1005618.s008.tif]

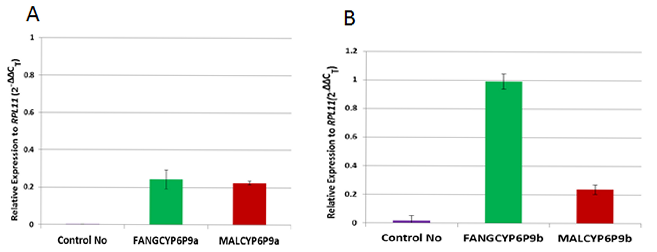

Supplement: S9 Fig — (TIF) [file pgen.1005618.s009.tif]

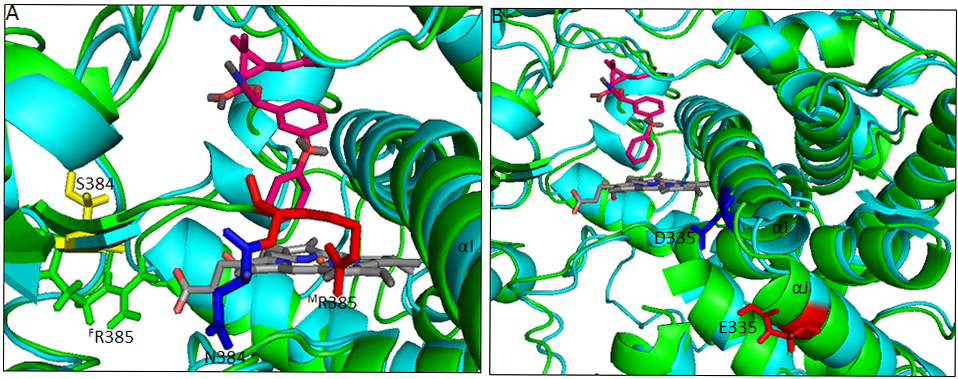

Supplement: S10 Fig — (A) FANGCYP6P9b-Ser384: yellow stick; FANGCYP6P9b-Arg385: green stick with superscript F; MALCYP6P9b-Asn384: blue stick; MALCYP6P9b-Arg385: red stick with superscript M; heme atoms: stick format and grey; deltamethrin in stick format and pink; (B) MALCYP6P9b-Asp335 is in stick form and blue colour, and FANGCYP6P9b-Glu335 is in red colour. (TIF) [file pgen.1005618.s010.tif]

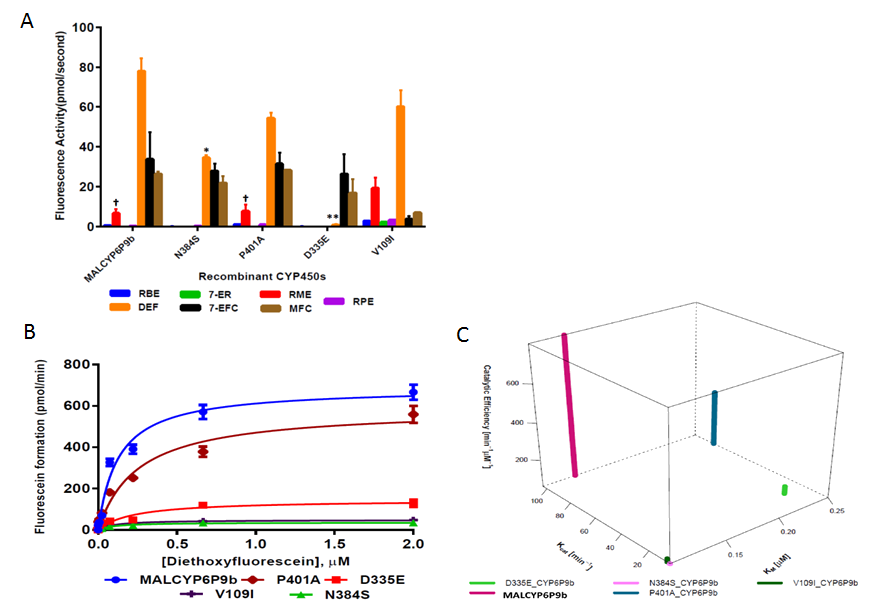

Supplement: S11 Fig — (A) O-dealkylation of seven fluorescent probes by the recombinant CYP6P9b mutants. The solid bars indicate average of significant activity in three experimental replicates compared to negative controls (-NADPH). RBE, resorufin benzylether; 7-ER, 7-ethoxyresorufin; RME, resorufin methylether; RPE, resorufin pentylether; DEF, diethoxyfluorescein; 7-EFC, 7-ethoxy-4-trifluoromethylcuomarin; MFC, 7-methoxy-4-trifluoromethylcuomarin. (B) Michaelis-Menten plots of mutant CYP6P9b proteins metabolism of diethoxyfluorescein; (C) 4D plot of the kinetic constants and catalytic efficiencies of mutant CYP6P9b proteins’ metabolism of diethoxyfluorescein. (TIF) [file pgen.1005618.s011.tif]
