# Supplementary material for: Allelic Variation of Cytochrome P450s Drives Resistance to Bednet Insecticides in a Major Malaria Vector
Source: PLoS Genet. 2015 Oct 30;11(10):e1005618. doi: 10.1371/journal.pgen.1005618 (PMC4627800; doi:10.1371/journal.pgen.1005618)
Supplement: S1 Table — (DOCX) [file pgen.1005618.s012.docx]

**S1 Table**: Summary statistics for polymorphism of *CYP6P9a* and *CYP6P9b* haplotypes across Africa

| **Samples** | **N** | **S** | **h** | **Syn** | **Nonsyn** | **π (k)** | **D (Tajima)** | **D* (Fu and Li)** |
| --- | --- | --- | --- | --- | --- | --- | --- | --- |
|  | ***CYP6P9a*** | | | | | | | |
| **Mozambique** | 5 | 5 | 4 | 0 | 5 | 0.001 (2.0) | -1.12^ns^ | -1.12^ns^ |
| **Malawi** | 6 | 7 | 5 | 2 | 5 | 0.0015 (2.3) | -1.3^ns^ | -1.4^ns^ |
| **Zambia** | 5 | 1 | 2 | 0 | 1 | 0.00039(0.6) | 1.2 ^ns^ | 1.2 ^ns^ |
| **Uganda** | 5 | 1 | 2 | 0 | 1 | 0.00026(0.4) | -0.82 ^ns^ | -0.82 ^ns^ |
| **Benin** | 6 | 34 | 4 | 32 | 2 | 0.0075(11.5) | -1.43 ^ns^ | -1.46 ^ns^ |
| **Fang** | 6 | 23 | 4 | 19 | 4 | 0.0076 (11.7) | -1.2^ns^ | -1.25^ns^ |
| **Total** | 33 | 74 | 17 | 52 | 22 | 0.014(21.9) | 0.76^ns^ | -0.12 ^ns^ |
|  | ***CYP6P9b*** | | | | | | | |
| **Mozambique** | 5 | 2 | 3 | 1 | 1 | 0.00079(1.2) | 1.45^ns^ | 1.45^ns^ |
| **Malawi** | 5 | 5 | 4 | 4 | 1 | 0.0013 (2.0) | -1.12^ns^ | -1.12^ns^ |
| **Zambia** | 5 | 1 | 2 | 0 | 1 | 0.00039(0.6) | 1.2 ^ns^ | 1.2 ^ns^ |
| **Uganda** | 5 | 1 | 2 | 0 | 1 | 0.00039(0.6) | 1.2 ^ns^ | 1.2 ^ns^ |
| **Benin** | 6 | 3 | 2 | 0 | 3 | 0.00065(1) | -1.23 ^ns^ | -1.23 ^ns^ |
| **Fang** | 5 | 38 | 4 | 31 | 7 | 0.01 (15.4) | -1.16^ns^ | -1.16^ns^ |
| **Total** | 31 | 138 | 11 | 87 | 51 | 0.029(44.6) | 0.94^ns^ | 1.42^*^ |

N= number of sequences (n); S, number of polymorphic sites; h, haplotype; Syn, Synonymous mutations; Nonsyn, Non-synonymous mutations; π, nucleotide diversity (k= mean number of nucleotide differences); Tajima’s D and Fu and Li’s D statistics, ns, not significant. * Statistically significant at p<0.05.
