# Supplementary material for: Allelic Variation of Cytochrome P450s Drives Resistance to Bednet Insecticides in a Major Malaria Vector
Source: PLoS Genet. 2015 Oct 30;11(10):e1005618. doi: 10.1371/journal.pgen.1005618 (PMC4627800; doi:10.1371/journal.pgen.1005618)
Supplement: S2 Table — (DOCX) [file pgen.1005618.s013.docx]

**S2 Table:** Key nucleotide polymorphisms and amino acid substitutions between sequences of CYP6P9a and CYP6P9b from resistant alleles compared with FANG

| **Amino acid substitution** | **Mutation** | **Countries** | **Location and potential Impact of mutation** |
| --- | --- | --- | --- |
| ***CYP6P9a*** | | | |
| **Ala^51^Ser** | 151:G->T | Benin and Uganda | Possibly located within the hydrophobic domain targeting the endoplasmic reticulum membrane |
| **Gln^52^Leu** | 153: C->T | Benin and Uganda | Possibly located within the hydrophobic domain targeting the endoplasmic reticulum membrane |
| **Phe^63^Leu** | 189: T->G | Malawi, Mozambique, Zambia | Within the highly variable αA region |
| **Gln^66^Lys** | 196: C->A | Malawi, Mozambique, Zambia | Within the highly variable αA region |
| **His^301^Gln** | 903: A->C | Uganda, Malawi, Mozambique, Zambia | Two residues upstream the substrate recognition site 4 (SRS-4) |
| **Tyr^320^Ser** | 959: A->C | Benin, Uganda, Malawi, Mozambique, Zambia | Middle of the αI helix and within SRS-4; one residue downstream the oxygen binding pocket (AGFETS) in CYP6P9a (Gotoh, 1992). |
| **Ser^431^Phe** | 1292: T->C | Benin, Uganda, Malawi, Mozambique, Zambia | Within the loop (16 residues in CYP6P9a) joining the meander with the cysteine pocket; the loop is purported to house the reductase interaction site 2 (RIS-2) (Hasemann et al., 1995). |
| ***CYP6P9b*** | | | |
| **Ser^32^Asn** | 95: G->A | Benin, Uganda, Malawi, Mozambique, Zambia | Within the hydrophobic residues anchoring the protein to membrane. May have impact on stability |
| **Ile^109^Val** | 325: A->G | Benin, Uganda, Malawi, Mozambique, Zambia | Within the SRS-1 and the BʹC loop purported to house residues involved in substrate access and channelling (Gotoh, 1992, Sirim et al., 2010). |
| **His^169^Arg** | 506: A->G | Benin, Uganda, Malawi, Mozambique, Zambia | C-terminus of D helix |
| **Gln^171^Pro** | 512: A->C | Benin, Uganda, Malawi, Mozambique, Zambia | N-terminus of the E helix |
| **Glu^172^Asp** | 516: G->T | Benin, Uganda, Malawi, Mozambique, Zambia | N-terminus of the E helix |
| **Glu^335^Asp** | 1005: A->C | Benin, Uganda, Malawi, Mozambique, Zambia | In the –NH_2_ terminus of the αJ helix, possible impact as RIS-1 residues mediating interaction with P450 reductase (Sirim et al., 2010). |
| **Ser^384^Asn** | 1151: G->A | Uganda, Malawi, Mozambique, Zambia | In the β1_4 (αKʹ) and within the highly conserved SRS-5, with possible impact on substrate recognition (Sirim et al., 2010, Gotoh, 1992). |
| **Ala^401^Pro** | 12001: G->C | Uganda, Malawi, Mozambique, Zambia | Within the β2_2 domain; pyrrolidine ring can restrict conformational space and affect chain rotations (MacArthur and Thornton, 1991). |
