# Supplementary material for: Allelic Variation of Cytochrome P450s Drives Resistance to Bednet Insecticides in a Major Malaria Vector
Source: PLoS Genet. 2015 Oct 30;11(10):e1005618. doi: 10.1371/journal.pgen.1005618 (PMC4627800; doi:10.1371/journal.pgen.1005618)
Supplement: S3 Table — (DOCX) [file pgen.1005618.s014.docx]

**S3 Table:** Binding parameters for permethrin and deltamethrin docked to the active sites of CYP6P9a and CYP6P9b models

| **Model** | **Pose Rank** | **ChemScore (kJ/mol)** | **ΔG**  **(kJ/mol)** | **S(hbond)** | **S(metal)** | **S(lipo)** | **ΔE(clash)** |
| --- | --- | --- | --- | --- | --- | --- | --- |
| **Permethrin** | | | | | | | |
| **MALCYP6P9a** | 2^nd^ | 44.72 | -43.49 | 0.00 | 0.00 | 356.63 | 0.15 |
| **FANGCYP6P9a** | 1^st^ | 37.91 | -38.91 | 0.00 | 0.00 | 317.47 | 0.90 |
| **BENCYP6P9a** | 2^nd^ | 44.14 | -45.60 | 0.00 | 0.00 | 374.66 | 0.24 |
| **UGANCYP6P9a** | 1^st^ | 45.77 | -47.54 | 0.00 | 0.00 | 391.27 | 1.50 |
| **MALCYP6P9b** | 1^st^ | 46.07 | -47.82 | 0.76 | 0.00 | 393.69 | 0.81 |
| **FANGCYP6P9b** | 1^st^ | 41.25 | -42.88 | 1.00 | 0.00 | 346.85 | 1.94 |
| **BENCYP6P9b** | 1^st^ | 43.44 | -44.22 | 1.01 | 0.00 | 362.91 | 0.22 |
| **UGANCYP6P9b** | 1^st^ | 43.32 | -44.04 | 0.21 | 0.00 | 332.92 | 0.23 |
| **Deltamethrin** | | | | | | | |
| **MALCYP6P9a** | 2^nd^ | 43.21 | -45.81 | 0.00 | 0.00 | 380.88 | 0.32 |
| **FANGCYP6P9a** | 5^th^ | 41.01 | -42.47 | 0.00 | 0.00 | 333.00 | 1.69 |
| **BENCYP6P9a** | 1^st^ | 43.31 | -46.31 | 0.00 | 0.00 | 384.88 | 0.79 |
| **UGANCYP6P9a** | 3^rd^ | 42.82 | -44.14 | 0.00 | 0.00 | 366.31 | 0.76 |
| **MALCYP6P9b** | 1^st^ | 45.90 | -49.76 | 0.49 | 0.97 | 364.36 | 0.23 |
| **FANGCYP6P9b** | 1^st^ | 39.77 | -41.38 | 1.97 | 0.00 | 307.33 | 0.98 |
| **BENCYP6P9b** | 1^st^ | 44.02 | -45.31 | 0.00 | 0.00 | 374.54 | 0.25 |
| **UGANCYP6P9b** | 1^st^ | 42.90 | -44.47 | 0.00 | 0.00 | 369.16 | 1.49 |

ΔG = free energy of binding, S (hbond) = contribution from hydrogen bonds, S (lipho) = lipophilic term, ΔE (clash) = clash penalties between ligand and receptors heavy atoms, and ΔE (int) = internal energy of the ligand or receptor.
