# Supplementary material for: Allelic Variation of Cytochrome P450s Drives Resistance to Bednet Insecticides in a Major Malaria Vector
Source: PLoS Genet. 2015 Oct 30;11(10):e1005618. doi: 10.1371/journal.pgen.1005618 (PMC4627800; doi:10.1371/journal.pgen.1005618)
Supplement: S4 Table — (DOCX) [file pgen.1005618.s015.docx]

**S4 Table:** Percentage depletion of pyrethroid insecticides by various recombinant CYP6P9a and CYP6P9b proteins

| **Recombinant Proteins** | **Permethrin** | **Bifenthrin** | **Deltamethrin** | **λ-cyhalothrin** | **Etofenprox** |
| --- | --- | --- | --- | --- | --- |
| **FANGCYP6P9a** | 29.0±1.42 | 16.6±5.41 | 18.11±2.15 | 15.46±4.43 | 7.28±1.53 |
| **UGANCYP6P9a** | 60.71±0.92* | 50.31±3.8* | 57.9±2.74* | 47.74±1.5* | 42.55±1.03* |
| **BENCYP6P9a** | 66.42±2.23* | 51.02±0.75* | 59.54±5.11* | 52.45±1.74* | 41.22±1.54* |
| **MALCYP6P9a** | 65.38±1.5* | 60.48±2.65** | 68.38±1.83** | 75.41±1.72* | 46.04±1.26* |
| **FANGCYP6P9b** | 13.7±4.23 | 22.38±5.08 | 6.2±1.5 | 15.49±3.13 | 16.88±3.06 |
| **UGANCYP6P9b** | 88.58±3.48** | 88.8±1.61** | 62.53±4.04** | 78.76±1.31** | 57.91±1.55* |
| **BENCYP6P9b** | 89.63±0.63** | 89.19±1.01** | 62.03±1.14** | 49.35±4.41* | 37.15±4.15* |
| **MALCYP6P9b** | 91.6±2.5** | 81.6±0.25** | 81.69±2.27** | 86.51±1.14** | 71.59±1.42** |

Values are mean ± S.D. of three replicates compared with negative control (-NADPH); * and ** significantly different from FANGCYP6P9a or FANGCYP6Pb at p<0.05 or p<0.01 respectively.
