# Supplementary material for: Allelic Variation of Cytochrome P450s Drives Resistance to Bednet Insecticides in a Major Malaria Vector
Source: PLoS Genet. 2015 Oct 30;11(10):e1005618. doi: 10.1371/journal.pgen.1005618 (PMC4627800; doi:10.1371/journal.pgen.1005618)
Supplement: S5 Table — (DOCX) [file pgen.1005618.s016.docx]

**S5 Tabl****e:** Kinetic constants for metabolism of permethrin and deltamethrin by various recombinant proteins of CYP6P9a and CYP6P9b

| **Recombinant proteins** | ***K_c_*_at_ (min^-1^)** | ***K_m_* (µM)** | ***K*_cat_/*K_m_* (min^-1^ µM^-1^)** |
| --- | --- | --- | --- |
|  |  | **Permethrin** |  |
| **BENCYP6P9a** | 9.37±3.32^a^ | 19.02±3.13^c^ | 0.49±0.19* |
| **UGANCYP6P9a** | 7.35±1.02 | 20.11±3.91^c^ | 0.37±0.08* |
| **FANGCYP6P9a** | 4.95±1.92 | 36.25±15.22 | 0.14±0.07 |
| **MALCYP6P9a** | 15.41±6.30^a^ | 18.77±11.76^c^ | 0.82±0.06* |
| **BENCYP6P9b** | 15.91±5.45^b^ | 21.94±7.97 | 0.73±0.36^$^ |
| **UGANCYP6P9b** | 8.61±3.40^b^ | 21.22±10.32 | 0.41±0.3^$^ |
| **FANGCYP6P9b** | 4.5±1.35 | 20.47±8.31 | 0.21±0.11^$^ |
| **MALCYP6P9b** | 12.38±4.5^b^ | 12.68±2.68^d^ | 0.97±0.41^$^ |
|  |  | **Deltamethrin** |  |
| **BENCYP6P9a** | 8.78±2.62^a^ | 15.67±4.64^c^ | 0.56±0.23* |
| **UGANCYP6P9a** | 7.96±3.62 | 14.48±3.58^c^ | 0.54±0.28* |
| **FANGCYP6P9a** | 4.87±2.06 | 24.03±3.12 | 0.20±0.08 |
| **MALCYP6P9a** | 14.65±4.12^a^ | 18.26±6.97 | 0.80±0.38* |
| **BENCYP6P9b** | 13.63±5.40^b^ | 15.98±5.63 | 0.85±0.45^$^ |
| **UGANCYP6P9b** | 10.44±4.00^b^ | 12.97±4.96^d^ | 0.80±0.35^$^ |
| **FANGCYP6P9b** | 4.92±0.82 | 19.5±5.53 | 0.25±0.08 |
| **MALCYP6P9b** | 12.09±1.44^b^ | 9.9±1.65^d^ | 1.22±0.25^$^ |

Values are a mean ±S.D. of three replicates.

Apparent *K_cat_* was calculated as pmol/min/pmol P450; Catalytic efficiency was calculated as *K*_cat_/*K****_m_***_._

^a,b^ Significantly different *K_cat_* values compared with FANGCYP6P9a and FANGCYP6P9b respectively, p<0.05.

^c,d^ Significantly different *K****_m_*** values compared with FANGCYP6P9a and FANGCYP6P9b respectively, p<0.05.

*^,$^ Significant differences between *K_cat_*/*K****_m_*** values respectively compared with FANCYP6P9a and FANGCYP6P9b*,* p<0.05.
