# Supplementary material for: Allelic Variation of Cytochrome P450s Drives Resistance to Bednet Insecticides in a Major Malaria Vector
Source: PLoS Genet. 2015 Oct 30;11(10):e1005618. doi: 10.1371/journal.pgen.1005618 (PMC4627800; doi:10.1371/journal.pgen.1005618)
Supplement: S6 Table — (DOCX) [file pgen.1005618.s017.docx]

**S6 Table:** Kinetic constants for recombinant CYP6P9a and CYP6P9b metabolism of diethoxyfluorescein

| **Recombinant Protein** | **Amount of Membrane (pmol)** | | ***K_m_* (µM)** | ***K_cat_* (min^-1^)** | ***K_cat_*/*K _m_* (min^-1^ µM^-1^)** |
| --- | --- | --- | --- | --- | --- |
| **BENCYP6P9a** | 10 | 0.091±0.006^a^ | | 14.16±0.23^c^ | 155.60±10.56* |
| **UGANCYP6P9a** | 10 | 0.226±0.035^a^ | | 12.98±0.61 | 57.43±9.29* |
| **FANGCYP6P9a** | 10 | 0.34±0.035 | | 9.52±0.33 | 27.99±3.04 |
| **MALCYP6P9a** | 10 | 0.14±0.013^a^ | | 22.84±2.21^c^ | 163.1±22.24* |
| **BENCYP6P9b** | 3.33 | 0.13±0.01^b^ | | 135.43±3.12^d^ | 1049.84±84.92^$^ |
| **UGANCYP6P9b** | 3.33 | 0.13±0.01^b^ | | 171.86±3.76^d^ | 1301.96±102.66^$^ |
| **FANGCYP6P9b** | 3.33 | 0.24±0.02 | | 14.93±0.500 | 62.20±5.58 |
| **MALCYP6P9b** | 3.33 | 0.12±0.01^b^ | | 156.27±3.306^d^ | 1220.85±98.81^$^ |

Values are mean ±S.D. of three replicates

Apparent *K_cat_* was calculated as pmol fluorescein produced/min/pmol P450; Catalytic efficiency was calculated as *K_cat_*/*K****_m_***_._

^a,b^ Significantly different *K****_m_*** values compared with FANGCYP6P9a and FANGCYP6P9b respectively. ^c,d^ Significantly different *K_cat_* values compared with FANGCYP6P9a and FANGCYP6P9b.

*^,$^ Significant differences between *K_cat_*/*K****_m_*** values respectively compared with FANCYP6P9a and FANGCYP6P9b.
