# Supplementary material for: Allelic Variation of Cytochrome P450s Drives Resistance to Bednet Insecticides in a Major Malaria Vector
Source: PLoS Genet. 2015 Oct 30;11(10):e1005618. doi: 10.1371/journal.pgen.1005618 (PMC4627800; doi:10.1371/journal.pgen.1005618)
Supplement: S7 Table — (DOCX) [file pgen.1005618.s018.docx]

**S7 Table:** Kinetic Constants for recombinant mutants of CYP6P9b metabolism of permethrin and diethoxyfluorescein

| **Recombinant Proteins** | ***K_cat_* (min^-1^)** | ***K_m_* (µM)** | ***K_cat_*/*K_m_* (min^-1^ µM^-1^)** |
| --- | --- | --- | --- |
|  | **Permethrin** |  |  |
| **Val^109^Ile_CYP6P9b** | 1.35±0.57** | 20.5±1.35^†^ | 0.06±0.03^$$$^ |
| **Asp^335^Glu_CYP6P9b** | 0.98±0.12*** | 5.19±1.89^†^ | 0.19±0.07^$$^ |
| **Asn^384^Ser_CYP6P9b** | 0.87±0.08*** | 17.85±0.87^†^ | 0.04±0.005^$$$^ |
| **Pro^401^Ala_CYP6P9b** | 4.21±0.96* | 15.38±6.50 | 0.27±0.13^$^ |
| **MALCYP6P9b** | 7.902±0.83 | 10.33±2.38 | 0.76±0.19 |
|  | **Diethoxyfluorescein** |  |  |
| **Val^109^Ile_CYP6P9b** | 7.32±0.13*** | 0.10±0.007 | 72.47±5.18^$$^ |
| **Asp^335^Glu_CYP6P9b** | 21.82±3.13** | 0.23±0.10† | 94.86±43.43^$$^ |
| **Asn^384^Ser_CYP6P9b** | 5.46±0.32*** | 0.10±0.023 | 54.05±12.71^$$^ |
| **Pro^401^Ala_CYP6P9b** | 88.32±7.57 | 0.25±0.07† | 353.28±103.44^$^ |
| **MALCYP6P9b** | 103.4±5.86 | 0.13±0.003 | 795.38±48.67 |

Values are as mean ± S.E.M. of three independent replicates. Significantly different from negative control (-NADPH).

Apparent *K_cat_* given as disappearance of permethrin/min/pmol P450. Catalytic efficiency was calculated as K*_cat_*/*K****_m._***

** and *** statistically significant *K_cat_* values at p<0.01 and p<0.001 respectively compared with MALCYP6P9b.

^$^ and ^$$^ statistically significant *K_cat_* values at p<0.05 and p<0.01 respectively compared with MALCYP6P9b.
