# Supplementary material for: Allelic Variation of Cytochrome P450s Drives Resistance to Bednet Insecticides in a Major Malaria Vector
Source: PLoS Genet. 2015 Oct 30;11(10):e1005618. doi: 10.1371/journal.pgen.1005618 (PMC4627800; doi:10.1371/journal.pgen.1005618)
Supplement: S8 Table — (DOCX) [file pgen.1005618.s019.docx]

**S8 Table:** List of primers used in this study

| **Gene/Allele** | | **Forward Primer** | | | | **Reverse Primer** | |  |
| --- | --- | --- | --- | --- | --- | --- | --- | --- |
| **Primers used for *CYP6P9a* and *CYP6P9b* *in vitro* functional characterisation** | | | | | | | | |
| **CYP6P9a_Full** | | ATGGAGCTCATTAACGTGGTGTTGGC | | | | | TCA CAA TTT TTC CAC CTT CAA GTA ATT ACC CGC |  |
| **CYP6P9b_Full** | | ATGGAGCTCATTAACGTGGTGTTGGC | | | | TTA CAC CTT TTC TAC CTT CAA GTA ATT ACC CGC | |  |
|  | |  | | | |  | |  |
| **ompA+2 Forward** | | GGAATTCCATATGAAAAAGACAGCTATCGCG | | | |  | |  |
| **ompA+2 CYP6P9aReverse** | | TCTAGAGAATTC TCACAATTTTTCCACCTTCAAG | | | |  | |  |
| **ompA+2 CYP6P9bReverse** | | TCTAGAGAATTC TTACACCTTTTCTACCTTCAAG | | | |  | |  |
| \| **Primers used for *in vivo* functional characterisation of *CYP6P9a* and *CYP6P9b* alleles: Transgenic analysis and qRT-PCR** \| \| \| \| \| \| --- \| --- \| --- \| --- \| --- \| \| **CYP6P9a_pUASattB** \| AGATCTATGGAGCTCATTAACGTGGTG \| \| TCTAGATCACAATTTTTCCACCTTCAAGTAA \|  \| \| **CYP6P9b_pUASattB** \| AGATCTATGGAGCTCATTAACGTGGTGTT \| \| TCTAGACTACAAAAACCCCTTCCGCT \|  \| \| **CYP6P9a_qRT-PCR** \| CAGCGCGTACACCAGATTGTGTAA \| TCA CAA TTT TTC CAC CTT CAA GTA ATT ACC CGC \| \|  \| \| **CYP6P9b_qRT-PCR** \| CAGCGCGTACACCAGATTGTGTAA \| TTA CAC CTT TTC TAC CTT CAA GTA ATT ACC CGC \| \|  \| \| ***RPL11*_qRT-PCR** \| CGATCCCTCCATCGGTATCT \| AACCACTTCATGGCATCCTC \| \|  \|   **Primers used for *in vitro* functional characterisation of *CYP6P9b*: site-directed mutagenesis** | | | | | | | | |
| ***MALCYP6P9a*_Leu^63^Phe_Lys^66^Gln** | | | | CTGGAA*T*T*T*TACAAA*C*AATTCAAGCAGCGCCGTG | | | CTTGAATT*G*TTTGTA*A*A*A*TTCCAGATGGATGTCGG | |
| ***MALCYP6P9a*_Gln^301^His** | GAATGACACA*C*CGAGAACTTGCGG | | | | GTTCTCG*G*TGTGTCATTCCTACTTC | | | |
| ***MALCYP6P9a*_Ser^320^Tyr** | GACATCAT*A*CACGACGCAAAGCTTC | | | | GCGTCGTG*T*ATGATGTCTCGAAACC | | | |
| ***MALCYP6P9a*_Phe^431^Ser** | GATCGCT*C*CTCACCGGAGGAAGTGAAG | | | | CTTCACTTCCTCCGGTGAG*G*AGCGATCC | | | |
| **MALCYP6P9b_Val^109^Ile** | GATCGCGGT*A*TTTTCACTAATGCAAG | | | | GTGAAAA*T*ACCGCGATCGTGG | | | |
| **MALCYP6P9b_Asp^335^Glu** | | | GAACCCTGA*A*ATCCAGGAGCGCCTTAG | | CCTGGAT*T*TCAGGGTTCTTTGCCAGC | | | |
| **MOALCYP6P9b_Asn^384^Ser** | | | GAATCGTTGA*G*TCGTGTGCCGTC | | CACGACT*C*AACGATTCTACCGGG | | | |
| **MALCYP6P9b_Pro^401^Ala** | | | CACGTGATT*G*CCAAACGAACGTTAG | | CGTTTGG*C*AATCACGTGTTTCG | | | |

Green is *EcoR*I, Purple is *Nde*I*,* Red is *Xba*I, and Blue is *Bgl*II*;* mutagenized positions in mutagenic primers are highlighted in pink and italicised.
