# Supplementary material for: Allelic Variation of Cytochrome P450s Drives Resistance to Bednet Insecticides in a Major Malaria Vector
Source: PLoS Genet. 2015 Oct 30;11(10):e1005618. doi: 10.1371/journal.pgen.1005618 (PMC4627800; doi:10.1371/journal.pgen.1005618)
Supplement: S1 Text — (DOCX) [file pgen.1005618.s020.docx]

**Supplementary Text**

**Methods**

**Amplification and cloning of *CYP6P9a* and *CYP6P9b* alleles for transgenic analysis**

Full length cDNA encoding *CYP6P9a* (*MALCYP6P9a* and *FANGCYP6P9a*) and *CYP6P9b* (*MALCYP6P9b* and *FANGCYP6P9b*) were separately amplified with forward and reverse primers (with *Bgl*II and *Xba*I restriction sites, respectively) using HotStarTaq Polymerase (QIAGEN) and as a template, the plasmidic pJET1.2 minipreps used for sequence characterisation. In a total volume of 15µl with final concentrations of 1X QIAGEN buffer (containing 15mM MgCl_2_), 0.8mM dNTP mixes, 0.22µM each of forward and reverse primers, 0.5-1.0µg of DNA template, 1U of HotStarTaq DNA polymerase and sterile water, amplification was carried out through initial denaturation of template at 95^o^C for 15 minutes, followed by 35 cycles each of 94^o^C for 30 seconds; 57^o^C for 30 seconds and 72^o^C for 90 seconds. This is then followed with final extension for 5 minutes at 72^o^C and hold at 4^o^C.

PCR product was cleaned and cloned into pJET1.2 blunt and transformed into *DH5α*. Positive colonies were mini-prepped overnight and sequenced on both strands. The minipreps were then double-digested with *Bgl*II and *Xba*I restriction enzymes, gel extracted, ligated into pUASattB vector already linearized with same restriction enzymes and transformed into *DH5α*. Medium scale plasmid preparation (Midiprep) was carried out using HiSpeed Plasmid Midi Kit (QIAGEN) according to manufacturer’s protocol. Midipreps were sent to Genetic Services, MA, USA (<http://www.geneticservices.com/>) for injection into flies. Using ФC31 system, clones were transformed into germline of a *D. melanogaster* strain carrying the attP40 docking site on chromosome 2 [“y1w67c23; P attP40”, “1; 2”]. Four transgenic lines, UAS-*MALCYP6P9a*, UAS-*FANGCYP6P9a*, UAS-*MALCYP6P9b* and UAS-*FANGCYP6P9b* were constructed. GAL4 lines were purchased from Bloomington Stock Centre (<http://flystocks.bio.indiana.edu/>). Ubiquitous expression of candidate genes in the transgenes in adult F_1_ progeny (the experimental group) was achieved after crossing homozygote males (UAS lines) with virgin females from the driver strain, Actin5C-GAL4 ["y [1] w[*]; P(Act5C-GAL4-w)E1/CyO","1;2"]. The experimental flies, with candidate gene (GAL4-UAS) exhibit red eyes, marker for UAS element, and normal wings from crossing with GAL4 driver. Flies with curly wings (UAS/CyO) devoid of the Actin5C-GAL4 driver were discarded. For control group, flies with the same background as the experimental group but devoid of the UAS and the candidate gene were crossed with the driver Actin5C-GAL4 lines to generate Actin5C-GAL4-*null* lines without insertion. Flies stock and the products of crossings were maintained at 25^o^C in plastic vials with food.

**Results**

***pw2a* channel lining residues in MALCYP6P9b and FANGCYP6P9b models**

For MALCYP6P9b, these tunnel-lining residues of which none is more than 3Å away from the tunnel include those from the SRS-1 (Arg^107^, Gly^108^, Val^109^, Thr^111^, Ser^119^, Leu^122^, Phe^123^, Leu^125^), those from SRS-2 (Phe^214^, Glu^215^, Leu^216^, and Asp^217^), residues from SRS-3 (Asp^245^, Val^246^ and Glu^247^), residues from SRS-4 and O_2_-binding pocket (Phe^316^, Ser^319^, Ser^320^ and Ser^324^), residues from SRS-5 including Arg^385^, Val^386^, Val^387^, Ser^388^, Asp^390^, Tyr^391^; residues from heme-binding region including Arg^452^, Val^453^, Cys^454^ (of the cysteine pocket which forms the 5^th^ axial ligand holding heme in place), as well as two residues from L-helix. These tunnel-lining residues from BC loop (Figure S5A), F/G loop, *β-1_4* (SRS-5), αI helix (SRS-4) correspond to the channel *pw2a* described for many cytochromes P450 [1, 2].

In the case of FANGCYP6P9b only 24 amino acid resides lined the tunnel gorge including those from SRS-1 (Arg^107^, Phe^110^, Thr^111^, Leu^118^, His^121^, Phe^123^ and Ala^124^); residues from the SRS-2 (Phe^214^, Glu^215^, Leu^216^), residues from FG loop (Phe^224^, Lys^240^, Ile^241^); one residue from SRS-3 (Phe^243^); residues from SRS-4 (including Gly^315^ and Thr^318^ of the oxygen-binding pocket, Phe^309^, Val^310^ and Leu^313^); Val^380^ of the SRS-5 as well as Cys^454^ of the cysteine pocket. Equally also, in the top ranked binding mode of deltamethrin in FANGCYP6P9b model the insecticide is not enshrouded by the tunnel in the active site of the P450, in contrast to the observation from the binding conformation of the deltamethrin in MALCYP6P9b model.

Differences in *pw2a* composition in *Helicoverpa zea* CYP321A1 and CYP6B8 result in the former P450 possessing spacious active site which binds and metabolizes bulky substrates more effectively, while the constrained active site of CYP6B8 results in bulky substrates binding further from the ferryl oxygen, for optimal metabolism [3]. For CYP6P9b the mutation Ile^109^Val possibly modifies the substrates accessing machinery in the resistant allele with impact on substrate recognition and/or affinity.

**Comparative assessment of pyrethroids (deltamethrin)-metabolising activities of various alleles of *CYP6P9a* and *CYP6P9b***

For *CYP6P9a*, highest turnover (*K_cat_*) was obtained from the southern African MALCYP6P9a, a maximal catalytic activity 3-fold higher than obtained from FANGCYP6P9a (p<0.05). BENCYP6P9a and UGANCYP6P9a also portrayed higher activities; with on average 2-fold higher *K_cat_* compared with FANGCYP6P9a (p<0.05). While no major difference was observed in the affinity (*K_m_*) between resistant southern African MALCYP6P9a and susceptible FANGCYP6P9a proteins*,* both BENCYP6P9a and UGANCYP6P9a exhibited higher affinity compared with FANGCYP6P9a (p<0.05). Despite the high *K_m_* exhibited by *MALCYP6P9a* its catalytic efficiency for deltamethrin was 4-fold higher than values obtained from FANGCYP6P9a (p<0.05), while values obtained from BENCYP6P9a and UGANCYP6P9a were on average 3-fold higher than from FANGCYP6P9a (p<0.05).

*For CYP6P9b,* membranes from all the resistant alleles metabolised deltamethrin with *K_cat_* on average more than 2-fold higher than maximal activity from FANGCYP6P9b-mediated activity (p<0.05) (Figure 3D and Table S5)*.* Significant differences were also observed in terms of affinity toward deltamethrin with the *K_m_* values exhibited by UGANCYP6P9b and MALCYP6P9b on average 2-fold lower than the *K_m_* from FANGCYP6P9b. Thus, MALCYP6P9b was calculated as 5-fold more efficient in deltamethrin metabolism compared with susceptible FANGCYP6P9b (p<0.05), while BENCYP6P9b and UGANCYP6P9b were also established as 3-fold more efficient than the recombinant protein FANGCYP6Pb from susceptible allele (p<0.05).

**qRT-PCR validation of expression of CYP6P9a and CYP6P9b in transgenic flies**

qRT-PCR confirmed that both *CYP6P9a* and *CYP6P9b* were expressed only in the transgenic F_1_ progenies from the crosses with Actin5C drivers and not expressed in the control flies (Figure S9). Assessment of the relative expression level of each allele for both genes showed that for *CYP6P9a* both transgenic flies expressing the resistant *MALCYP6P9*a and the susceptible *FANGCYP6P9a* had a similar expression level. For *CYP6P9b* a higher over-expression was observed in the transgenic flies expressing the susceptible allele (progeny of crosses between Actin5C-GAL4 and UAS-*FANGCYP6P9b*) with a four-fold higher expression compared to the flies expressing the resistant allele (progeny from crosses between Actin5C-GAL4 and UAS-*MALCYP6P9b)*. Therefore, the significantly higher resistance observed in flies over-expressing the resistant alleles is not due to a higher expression of these alleles but rather caused by the allelic variation which appears to be pre-eminent over over-expression.

**Pattern of expression of mutant CYP6P9b recombinant proteins**

The Val^109^Ile mutant was expressed using *E. coli* *DH5α* by lowering the orbital shaking to 120 rpm after induction of the log-phased cells with 0.5mM δ-ALA and 0.5mM IPTG to the final concentration. With the exception of Pro^401^Ala all the mutants expressed lower amount of functional P450s compared with the wild type *MALCYP6P9b*. Specifically, the Val^109^Ile mutant expressed with concentrations of less than 1.0nmol/ml in all the three successful attempts. Asp^335^Glu, Asn^384^Ser and Pro^401^Ala expressed with concentrations of 2.97±0.47nmol/ml, 2.77±0.41nmol/ml and 5.31±1.81nmol/ml, respectively, compared with 4.89±0.46nmmol/ml from the wild type MALCYP6P9b. Time to optimal expression for all the mutants is between 40-48 hours with the exception of Pro^401^Ala mutant which expresses optimally before 30 hours after induction and the Val^109^Ile mutant which expressed slowly but optimally at around hours.

**Spatial distribution of key residues in the active site of MALCYP6P9b and FANGCYP6P9b models**

The Ser^384^ in FANGCYP6P9b is located within the *β-1_4* placing the guanidinium group of Arg^385^ away (within 9.6Å from 4ʹ spot of pyrethroid substrate) (Figure 2B, Figure S10A). In contrast, the peptide bond between Asn^384^ and Arg^385^ in MALCYP6Pb (~120^o^) positioned both Asn^384^ and Arg^385^ within the loop joining *β-1_4* with *β-2_1*. The amido group of Asn^384^ is thus situated within 6.6Å distance from guanidinium group of Arg^385^ which in turn is within 6.9Å of the 4ʹ spot of the phenoxy group of oriented towards the heme. These subtle difference may account for the reason why Arg^385^ is a *pw2a* tunnel lining residue in MALCYP6P9b and thus involved in substrate accessing the heme catalytic centre, while the corresponding residue is absent in *pw2a* from FANGCYP6P9b. In MALCYP6P9b the Asn^384^-Arg^385^ bond positioned guanidinium moiety of Arg^385^ within 5Å distance of Ser^324^. It is assumed that catalysis may be effected in MALCYP6P9b through hydrogen bonding network involving polar residues arrayed round the binding site and distal to the heme, including Asn^384^ and Arg^385^ (SRS-5), Arg^452^ and Cys^454^ (heme-binding region), Asn^502^, Asp^217^ (SRS-2), Ser^320^ (O_2_-binding pocket), Ser^324^ as well as Gln^323^ (αI). Presence of these protonation machineries in the distal side of the heme could accelerate the rate of conversion of Compound 0 to Compound I enhancing rate of catalysis and reducing the formation of undesirable O_2_-wasting species. In contrast, in the productive pose of deltamethrin in FANGCYP6P9b, residues Ser^384^ and Arg^385^ located within the *β-1_4* are not within 9.0Å of deltamethrin or heme.

The same pattern of differences in the backbone folding of the models of CYP6P9b affected the positioning of residue 335. In this respect, Glu^335^ residue in the FANGCYP6P9b is located in the *N*-terminus of αJ helix and within a distance of 30.7Å from the heme (Figure 2, Figure S10B), while the smaller Asp^335^ in MALCYP6P9b is positioned in the *C*-terminus of helix I and within 20.3Å from heme. Glu^335^ in FANGCYP6P9b mapped to the putative reductase interaction site 1 (RIS-1) [4] and its assumed that the presence of this residue in the αJ helix may result in ionic repulsion with the corresponding negatively charged residues at the Flavin mononucleotide (FMN) face of cytochrome P450 reductase, reducing optimal interaction with the redox partner and by that the overall catalysis.

Each of the residues in MALCYP6P9b implicated as critical for activity is assumed to contribute towards catalysis either through channelling and/or specificity (Val^109^ and Asn^384^), hydrogen bonding network (Asn^384^ through proper positioning of Arg^385^) or interaction of the proximal residues with cytochrome P450 reductase (absence of acidic side chain of Asp^335^ in the RIS-1 which optimizes interaction with the FMN binding domain).

***Fluorogenic probes assays with mutant membrane of MALCYP6P9b***

Seven probe substrates were tested for O-dealkylation activity with recombinant proteins expressed from *MALCYP6P9b* mutants. Highest activity was obtained with diethoxyfluorescein (DEF) and lowest activity with the resorufin-based substrates, though Val^109^Ile, Pro^401^Ala and MALCYP6P9b proteins exhibited quantitative increase in activity toward RME (Figure S11A). The reduction in activity of the mutant membranes with DEF is most profound with Asp^335^Glu mutant (25-fold reduction, p<0.001) as well as the Asn^384^Ser mutant (2-fold reduction, p<0.05) compared with the wild type MALCYP6P9b. The Asp^335^Glu and Asn^384^Ser mutants exhibited almost no detectable enzymatic activity toward all the resorufin-based probes.

The kinetic parameters of MALCYP6P9b-mediated O-dealkylation of DEF differed significantly from that of the mutant alleles. Fluorescein formation follows Michaelis-Menten pattern (Figure S11B) and in the case of MALCYP6P9b and Pro^401^Ala proceeds with high turnover roughly 5-fold than values obtained with the Asp^335^Glu mutant, 14-times the maximal activity observed with Val^109^Ile mutant, and 19-fold higher than maximal activity obtained with Asn^384^Ser mutant (Figure S11C and Table S7). Asp^335^Glu and Pro^401^Ala replacement doubled the *K_m_* of DEF, while no significant shift in *K_m_* was observed in Val^109^Ile and Asn^384^Ser mutants. The catalytic efficiency of the wild type *MALCYP6P9b* is established as more than 8-fold, 10-fold and 15-fold greater than values from Asp^335^Glu, Val^109^Ile and Asn^384^Ser mutants, respectively, indicating that the three amino acids replaced (Val^109^, Asp^335^ and Asn^384^) in southern African allele *MALCYP6P9b* are critical for optimal activity and/or affinity toward DEF.

**Discussion**

***Evidences from variation in activity towards pyrethroids and probe substrates:***

The *K_m_* values obtained for both permethrin and deltamethrin from resistant alleles of *CYP6P9a* and *CYP6P9b* were also very close to the *K_m_* values established for *An. gambiae* CYP6M2 with permethrin (12.0µM) and higher than with deltamethrin (2.0µM) [5] and lower than the *K_m_* values from metabolism of pyrethroids by *An. minimus’* CYP6P7 and CYP6AA3 [6]. Nevertheless, the differences observed in activities resulted in a very high catalytic efficiency in the resistant alleles of *CYP6P9a* and *CYP6P9b*, especially those from southern Africa compared with *FANGCYP6P9a* and *FANGCYP6P9b*. For example, MALCYP6P9b protein exhibited efficiencies of 0.97min^-1^µM^-1^ and 1.22min^-1^µM^-1^ respectively for permethrin and deltamethrin compared with FANGCYP6P9b protein (catalytic efficiencies of 0.21min^-1^µM^-1^ and 0.25min^-1^µM^-1^), *Anopheles gambiae* CYP6M2 (~0.5min^-1^µM^-1^ for both pyrethroids) [5] or *Anopheles minimus* CYP6P7 and CYP6AA3 (0.75min^-1^µM^-1^ for deltamethrin) [6]. This established that *CYP6P9a* and *CYP6P9b* alleles from resistant strains (especially those from southern Africa) are efficient metabolisers of pyrethroids (permethrin and deltamethrin) compared with corresponding enzymes from the susceptible strain (*FANGCYP6P9a* and *FANCYP6P9b*), and also compared with other insect 450s reported from other studies.

Furthermore, the difference between resistant and susceptible alleles of these genes also extended to their of O-dealkylation activity on fluorogenic probes as a significantly higher activity was observed for resistant alleles particularly with DEF. The *K_m_* values obtained from proteins expressing the resistant alleles of both C*YP6P9a* and *CYP6P9b* were comparable to the *K_m_* established for *An. gambiae* CYP6Z2- and *Aedes aegypti* CYP6Z8-mediated de-benzylation of benzyloxyresorufin (*K_m_* ~0.13µM) [7, 8], while the *K_m_* from FANGCYP6P9a and FANGCYP6P9b are 3-fold and 2-fold higher than these values, respectively. The dealkylation of DEF by the resistant alleles proceeds with high turnover, greater than established for some probes with insect P450s; for example *An. gambiae* CYP6Z2 with benzyloxyresorufin (*K*_cat_ ~1.5min^-1^) [7] and *Ae. aegypti* CYP6Z8 with benzyoxyresorufin and ethoxyresorufin (0.097min^-1^ and 1.19min^-1^ respectively) [8]. This resulted in higher catalytic efficiency obtained from proteins expressing the resistant alleles of *CYP6P9a* and *CYP6P9b,* compared with *FANGCYP6P9a* and *FANGCYP6P9b* and compared to the values established from the studies cited above. The alleles of *CYP6P9a* and *CYP6P9b* from resistant strains are in essence significantly more efficient as probe substrate metabolisers compared with the susceptible alleles. Allelic variation is thus impacting on enzymatic activity of the resistant alleles towards fluorescent probes, modifying kinetic constants and efficiency of O-dealkylation of the probe substrates.

***Evidences from transgenic analysis***

Expression of resistant alleles alone confers resistance to pyrethroids suggesting that allelic variation is even more important than over-expression. However, it remains also to establish the importance of gene over-expression compared with allelic variation observed in this study. Over-expression is obviously important for higher turnover of a particular candidate gene, which can make it more abundant to detoxify insecticides. However the inability of the susceptible alleles of both *CYP6P9a* and *CYP6P9b* to confer resistance even when over-expressed, suggests that allelic variation through replacement of key residues, can further increase detoxification activity via several mechanisms which can modify specificity and/or catalytic activity toward substrates. This is also similar to the case of the *GSTe2* gene in *An. funestus* for which the presence of the Leu^119^Phe resistance mutation was associated with up-regulation of this gene in Benin while in region of low expression the mutation was also absent [9].

***Key amino acid changes control pyrethroid resistance and could lead to DNA-based diagnostic tools***

Leu^209^Ala replacement in rat *CYP2B1* has been established to result in qualitative changes in activity allowing progesterone to bind in a new orientation [10]. The larger Leu hinders the substrate from assuming productive orientation due to vdW overlaps. This maybe the case in Val^109^Ile mutant of *CYP6P9b* in which the smaller side chain of valine is replaced with the larger isoleucine the latter residue which possibly repels the pyrethroid insecticides through vdW overlap.

Also, Val^92^Ala replacement in *Depresseria pastinacella* *CYP6AB3v1* makes this allelic variant capable of metabolising plant allelochemical imperatorin nearly as effective as *CYP6AB3v2* furanocoumarins metaboliser [11]. Replacement of even a single amino acid residue can have profound effect on topology of active site, specificity and overall activity towards particular substrate or broad range of substrates. Recently, in *GSTe2* a single amino acid replacement (Leu^119^Phe) was established as the sole reason for very high DDT resistance in *An. funestus* population from Benin, West Africa [9], setting a pace for the quest of markers of metabolic resistance in mosquito vectors.

**References**

1. Wade RC, Winn PJ, Schlichting I, Sudarko. A survey of active site access channels in cytochromes P450. Journal of inorganic biochemistry. 2004;98(7):1175-82. doi: 10.1016/j.jinorgbio.2004.02.007. PubMed PMID: 15219983.

2. Cojocaru V, Winn PJ, Wade RC. The ins and outs of cytochrome P450s. Biochimica et biophysica acta. 2007;1770(3):390-401. Epub 2006/08/22. doi: S0304-4165(06)00205-4 [pii]

10.1016/j.bbagen.2006.07.005. PubMed PMID: 16920266.

3. Rupasinghe SG, Wen Z, Chiu TL, Schuler MA. Helicoverpa zea CYP6B8 and CYP321A1: different molecular solutions to the problem of metabolizing plant toxins and insecticides. Protein engineering, design & selection : PEDS. 2007;20(12):615-24. Epub 2007/12/11. doi: 10.1093/protein/gzm063. PubMed PMID: 18065401.

4. Sirim D, Widmann M, Wagner F, Pleiss J. Prediction and analysis of the modular structure of cytochrome P450 monooxygenases. BMC structural biology. 2010;10:34. doi: 10.1186/1472-6807-10-34. PubMed PMID: 20950472; PubMed Central PMCID: PMC3224734.

5. Stevenson BJ, Bibby J, Pignatelli P, Muangnoicharoen S, O'Neill PM, Lian LY, et al. Cytochrome P450 6M2 from the malaria vector Anopheles gambiae metabolizes pyrethroids: Sequential metabolism of deltamethrin revealed. Insect Biochem Mol Biol. 2011;41(7):492-502. Epub 2011/02/18. doi: 10.1016/j.ibmb.2011.02.003. PubMed PMID: 21324359.

6. Duangkaew P, Pethuan S, Kaewpa D, Boonsuepsakul S, Sarapusit S, Rongnoparut P. Characterization of mosquito CYP6P7 and CYP6AA3: differences in substrate preference and kinetic properties. Archives of insect biochemistry and physiology. 2011;76(4):236-48. Epub 2011/02/11. doi: 10.1002/arch.20413. PubMed PMID: 21308761.

7. McLaughlin LA, Niazi U, Bibby J, David JP, Vontas J, Hemingway J, et al. Characterization of inhibitors and substrates of Anopheles gambiae CYP6Z2. Insect Mol Biol. 2008;17(2):125-35. Epub 2008/03/21. doi: IMB788 [pii]

10.1111/j.1365-2583.2007.00788.x. PubMed PMID: 18353102.

8. Chandor-Proust A, Bibby J, Regent-Kloeckner M, Roux J, Guittard-Crilat E, Poupardin R, et al. The central role of mosquito cytochrome P450 CYP6Zs in insecticide detoxification revealed by functional expression and structural modelling. The Biochemical journal. 2013;455(1):75-85. doi: 10.1042/BJ20130577. PubMed PMID: 23844938; PubMed Central PMCID: PMC3778711.

9. Riveron JM, Yunta C, Ibrahim SS, Djouaka R, Irving H, Menze BD, et al. A single mutation in the GSTe2 gene allows tracking of metabolically based insecticide resistance in a major malaria vector. Genome Biol. 2014;15(2):R27. doi: 10.1186/gb-2014-15-2-r27. PubMed PMID: 24565444; PubMed Central PMCID: PMC4054843.

10. Szklarz GD, He YA, Halpert JR. Site-directed mutagenesis as a tool for molecular modeling of cytochrome P450 2B1. Biochemistry. 1995;34(44):14312-22. PubMed PMID: 7578035.

11. Mao W, Rupasinghe SG, Zangerl AR, Berenbaum MR, Schuler MA. Allelic variation in the Depressaria pastinacella CYP6AB3 protein enhances metabolism of plant allelochemicals by altering a proximal surface residue and potential interactions with cytochrome P450 reductase. J Biol Chem. 2007;282(14):10544-52. Epub 2007/01/25. doi: 10.1074/jbc.M607946200. PubMed PMID: 17244619.
